# Effect of explosives charges types on the jet characteristics, penetration performance and fragmentation patterns of shaped charges

**DOI:** 10.1038/s41598-024-75727-0

**Published:** 2024-11-01

**Authors:** Tamer Elshenawy, Gamal M. Abdo, Ahmed Elbeih

**Affiliations:** 1 Technical Research Center, Cairo, Egypt; 2 Mechanical Engineering department, Faculty of Engineering and Technology, Badr University, Cairo, Egypt; 3 Military Technical College, Kobry Elkobbah, Cairo, Egypt

**Keywords:** Plastic bonded explosives, HMX, Shaped charges, Penetration, Fragmentation, Flash X-ray radiograph, Autodyn, Engineering, Materials science

## Abstract

Different explosive materials have been studied numerically and experimentally to assess the efficiency of a small diameter shaped charge in terms of produced jet characteristics and penetration depth into RHA steel targets. 26 different explosives have been simulated numerically using Autodyn hydrocode, whereas recommended explosives have been loaded into small diameter shaped charges by pressing technique and tested by static firing against RHA targets in order to validate the numerical calculations. The numerical analysis has presented an intensive global view about the variation of the shaped charge jets as a potential of the loaded explosive charge efficiencies. A successful trial has been performed to measure the shaped charge jet velocity using detonation velocity VOD 812 apparatus, where its measured value was only 3.6% different from the numerical one for HMX-V5 explosive. Besides, TITAN (L3) flash X-ray radiograph has also been implemented to explore the jet profile using the same explosive type and to measure its jet tip velocity, which has only 2.1% different from that estimated numerically. Extensive fragmentation analysis has been presented, which showed increase in both the fragment number and the fragment speed when the used explosive charge is of high detonation velocity. CL-20 explosive exhibited the largest jet tip velocity and its scaled collapse velocity was found to be 140% of TNT explosive. The calculated average fragment speed has been validated and the measured fragment speed has only 2.3% difference when compared to the SPH calculations.

## Introduction

Shaped charge device produces hypervelocity jet that has high penetration efficiency into different targets depending on the jet mass, velocity, symmetry and its ductility^1–3^. Several researches have been conducted to study the dependence of the produced jet characteristics on the well-known various parameters^4–6^. The liner material type, its crystallographic structure and its grain size have been discussed in details in reference^7^. Influence of the jet temperature and its grain size (below 5 to 100 μm) on its breakup and subsequent predicted penetration potential was discussed in literature^8,9^. The effect of the explosive load types has been discussed^10^, while the liner shape design^11–13^ and their effect on the produced jet characteristics and relevant penetration depth have been investigated and addressed. Theoretically, more energetic explosives produce fast jet, greater jet kinetic energies and more depth of penetration^14^. The explosive density, the presence of air bubbles and cracks inside the explosive also affect the shaped charge penetration ability into the targets^15^. Pressing of the explosives should be undertaken on vacuum to drive out the air bubbles and to obtain high density of charges as discussed by Renfre et al.^16^. In addition, flash x-ray is usually used for checking the air voids and cracks in the military explosive charges. Other parameters including the explosive particle sizes and its homogeneity have to be checked^14^. Moreover, it has been supposed that the shaped charge warhead may be expected to produce high penetration efficiency in case of charges filled by particle sizes less than 200 μm^17^.

Many researchers have shown explicit dependence of the shaped charge jet characteristics and relevant achieved penetration depth and crater volume on the detonation characteristics of the used explosive. Michael et al.^18^ showed that the powerful LX-19 explosive was prepared by coating the CL-20 crystals with estane binder. The grain size of the CL-20 crystals has been optimized so that the theoretical maximum density can be approached. They have discussed some experimental testing of shaped charges, fragmentation charges and explosive formed projectiles using the two powerful plastic bonded explosives based on CL20 and HMX. The studied shaped charges have a trumpet liner and loaded with three different explosives, which are Octol, LX-14 and LX-19. There was a remarkable increase in the penetration depth at all the stand-off distances when the powerful LX-19 explosive was loaded in comparison with Octol and LX-14. They also had a promising result of the explosive LX-19 when used instead of A-3 in a dual purpose (fragmentation and penetration) of small shaped charges. The powerful LX-19 explosive increased the fragment velocity by 7% and the number of fragments obtained from the charge casing by 71%, whereas the penetration depth was increased by only 2% in comparison with the baseline A-3 explosive.

Elbeih et al. have studied the explosive characteristics of several advanced explosives, which might be candidate for the applications of shaped charges^19–21^. BCHMX is an interesting advanced energetic material with sensitivity in the range of PETN^22^ and its performance is in the range of HMX^23^. In addition the thermal reactivity of several advanced explosives in comparison with BCHMX has been discussed in literature^24–26^. The application of small calibre shaped charges loaded with different cyclic nitramines including RDX, HMX, CL20 and BCHMX have been tested^27^. The results showed different penetration depths into RHA targets where the largest penetration depth was achieved with the CL-20-based PBX with 20% larger than that of the RDXbased PBX.

Moser et al.^28^ have tested several formulations based on TNAZ and CL20 explosives in comparison with 95.5% HMX as a baseline explosive charge. They found that using more energetic explosives such as TNAZ and CL20 produced larger EFP with higher velocity, which in turn achieved 5–9% larger penetration depth in comparison to the baseline LX-14 explosive. Besides, the optimized formulations based on TNAZ and CL20 explosives have revealed 20–30% increase in the penetration depth compared to the baseline LX-14 explosive. The effect of the explosive type on the collapse, stagnation and relevant jet velocity was also studied numerically and experimentally by Stanley et al.^29^. They have used few explosives and cone angle configurations to find out the relation between flow, stagnation and jet tip coherent velocity using three explosives; LX-14, LX-19 and Octol. They found various collapse and flow velocities depended mainly on the used explosives, which in turn have a direct impact on the jet tip velocity. Elshenawy et al.^30^ showed that using various explosives can yield different jetting analysis data including jet velocities and characteristic stagnation point –distance histories for stretching jet with each studied explosive, which results in different locations in the virtual origin point and their varied penetration depth estimation accordingly.

All the above mentioned research revealed the rule of the chemical energy and its accompanied effect during the implosion of the liner element and the jet formation. However, few researches have been conducted on driving the shaped charge liner using alternative energy form such as electromagnetic energy. It has been proved that the electromagnetic energy from mega amperes electric capacitor is able to collapse the copper liner and produce a realistic jet^31^. Fred et al. have designed small, intermediate and large scale special liners that have been tested to collapse and form jet without using energetic high explosive. They have been accelerated under high ampere as much as 8.78 mega ampere peak current. Although the three tested accelerated liners have different mechanisms than the traditional liners loaded by shock explosive charge, they have achieved reasonable jets with some reasonable penetration tests into steel targets.

The main aim of the study is to check the dependence of small diameter shaped charges jet tip velocities and their penetration potential on the type of the loaded explosive charge including advanced explosives such as BCHMX and CL-20. The first part includes the hydrodynamic numerical calculations of the shaped charge jet parameters using Autodyn, after which the evolved jet is allowed to penetrate into RHA targets, where its penetration depth is estimated. Similarly, the smooth particle hydrodynamic (SPH) algorithm built in Autodyn has been used to study the variation in both the fragment masses and velocities with different loaded explosives. Besides, experimental measurements based on HMX have been used to validate the penetration testing of these shaped charges. Moreover, the VOD 812 apparatus by OZM research has been used to measure the shaped charge jet velocity and to validate the used hydrocode.

## Experimental work

### Shaped charge assembly and static firing

**Fig. 1:**
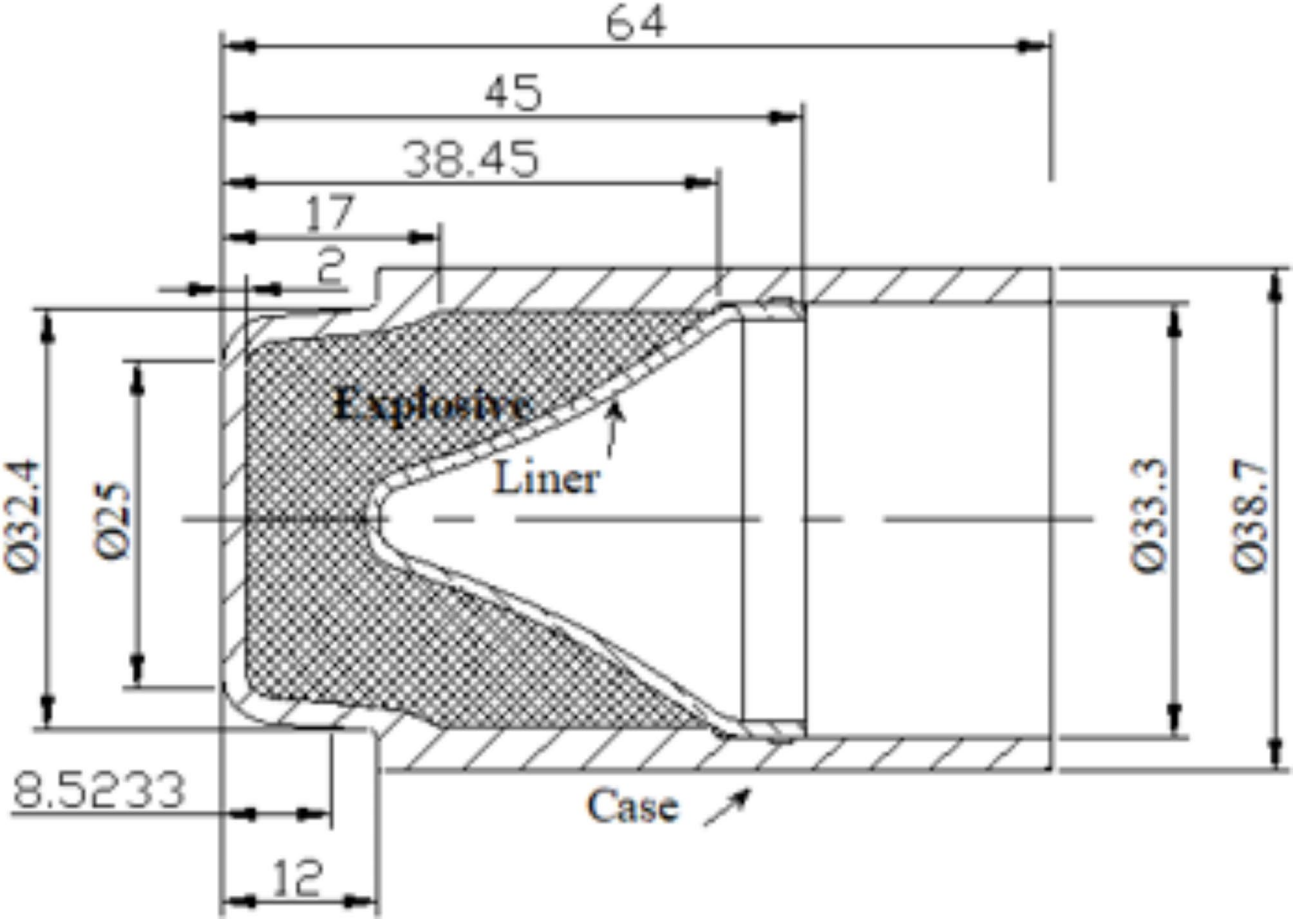
The studied shaped charge.

The used liners with small caliber were manufactured by spinning technique using CNC 500 shear forming machine produced from DENN Company, Spain. The copper liners are produced from electrolytic oxygen-free copper (99.99% purity), that has a suitable ductility and machinability with a 1 mm thickness of liner wall. The liner has a base diameter of 31.8 mm, height of 27.35 mm and of trumpet shape as shown in Fig. 1. The initial thickness of the copper sheet was 3 mm, where the liner is obtained by spinning with an attenuation of the wall to the designed liner thickness. The obtained liners showed a structure with grain size: 15–25 micrometers and hardness: 55–65 HV5.

**Fig. 2:**
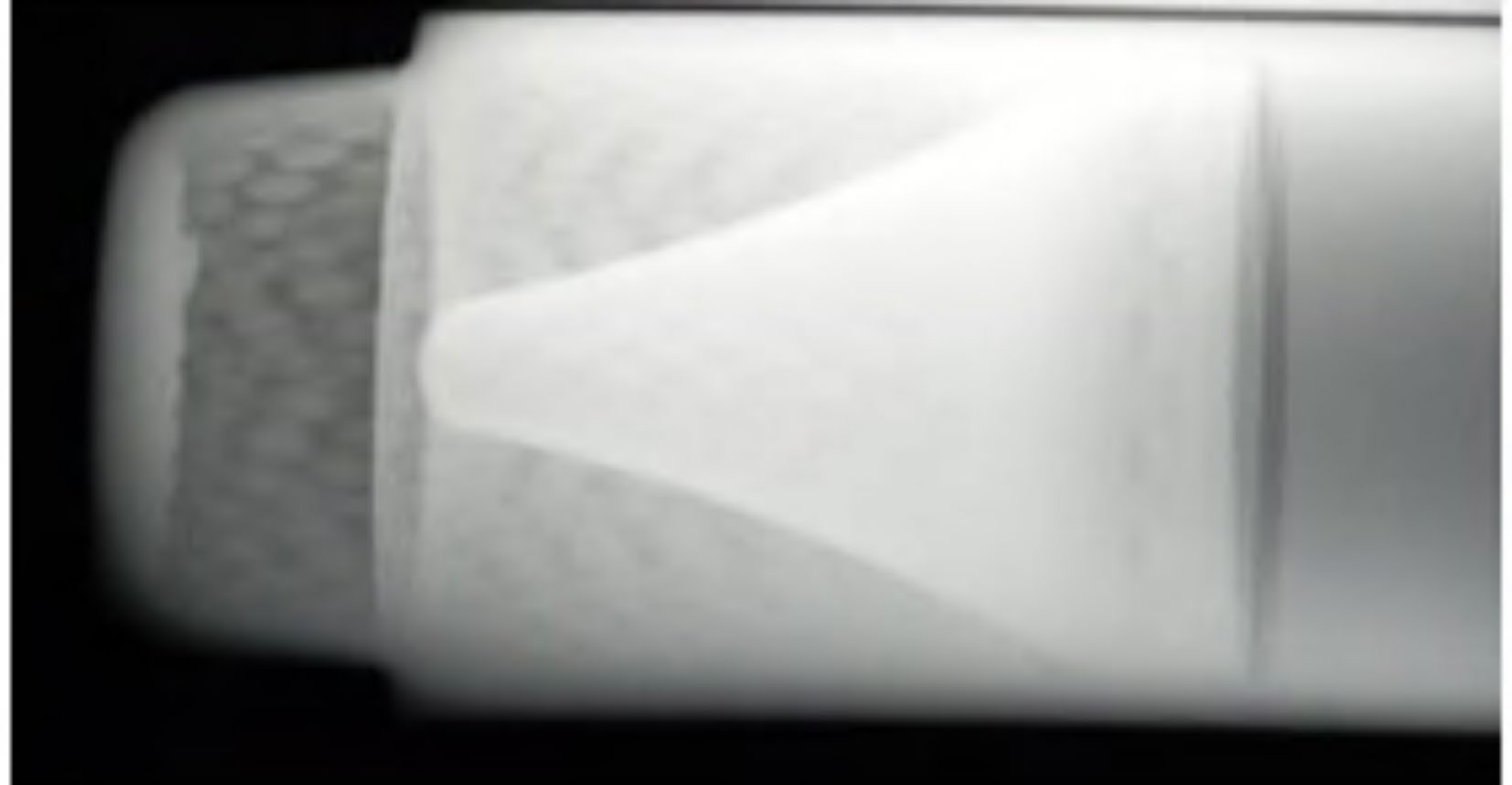
X-ray photograph of the explosive charge.

The case of the charge case is made from steel 1006 sheet using a deep drawing technique. The external case diameter is 38 mm, and the thickness of its wall is 2 mm. The explosive charges were produced in our department and composed of HMX 95 wt% and Viton A 5 wt%. The preparation method is based on the slurry technique as discussed by elbeih et al.^30^. The filled explosive charge mass is 40 g pressed inside the charge case during three stages by applying 50 ton pressing force for 10 s dwell time. The explosive charge was heated at 60 ^o^C before applying the pressing conditions to remove the humidity and enhance the pressing density. Static X-ray photograph has been taken to figure out there is no any air voids or cracks inside the explosive charge as depicted in Fig. 2. The experimental test is based on placing the shaped charge at 30 mm (i.e. 1D sand-off distance) above the rolled homogenous armor (RHA) and firing it using Briska electric detonator. High speed camera had been used to determine the fragment velocity of the steel body during the static firing of the shaped charge. Photron FASTCAM NOVA S12 high speed digital imaging system has been used with frame rate of 500k frame per second.

### Flash x-ray radiograph

**Fig. 3:**
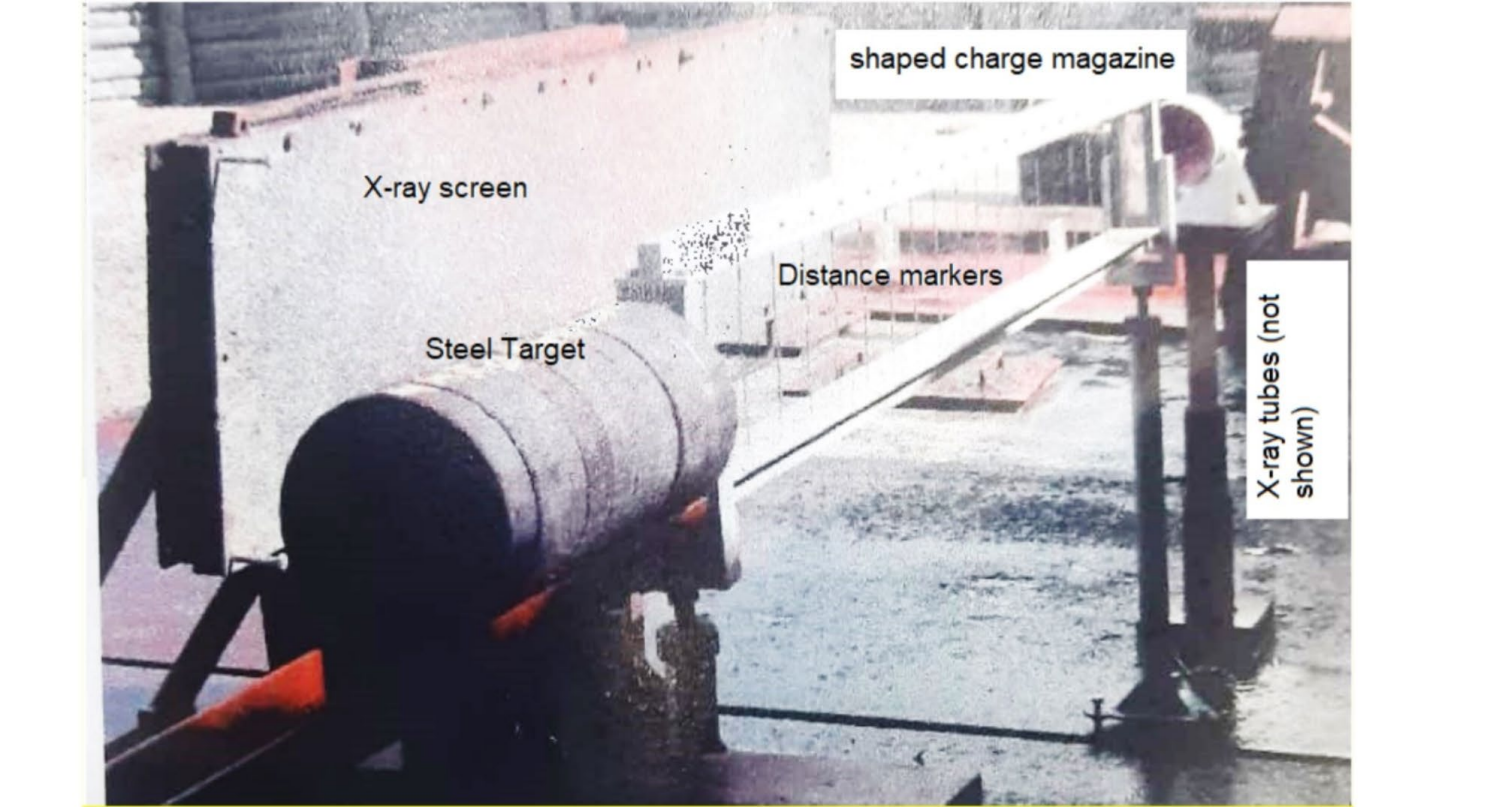
The flash x-ray trial setup.

In addition to Autodyn hydrocode validation, the shaped charge studied in the current research as a validation test, has been photographed by two head tubes of 300kv and 1MV flash X-ray radiograph. The flash x-ray is used to measure the jet tip velocity and to predict the jet profile at different times to validate the numerical results in the current study with respect to the jet tip velocity and the jet profile. The flash x-ray trial was performed using two heads supplied by Titan’s (upgraded to L3 company) facility to capture photos of the jet profile at different times. Figure 3 shows the setup of the x-ray trial field test. The initial delay times at which the x-ray trial photos have been set were. 13, 24, 26, 38µs for continuous jet; whereas particulation times of 150 and 220µs.

The jet tip velocity was found 2.1% different from that of the numerical simulation. (V_tip_-x-ray = 9160 m/s). Unfortunately we couldn’t have more verification measurements of the jet tip velocity due to its high cost.

## Numerical model

**Fig. 4:**
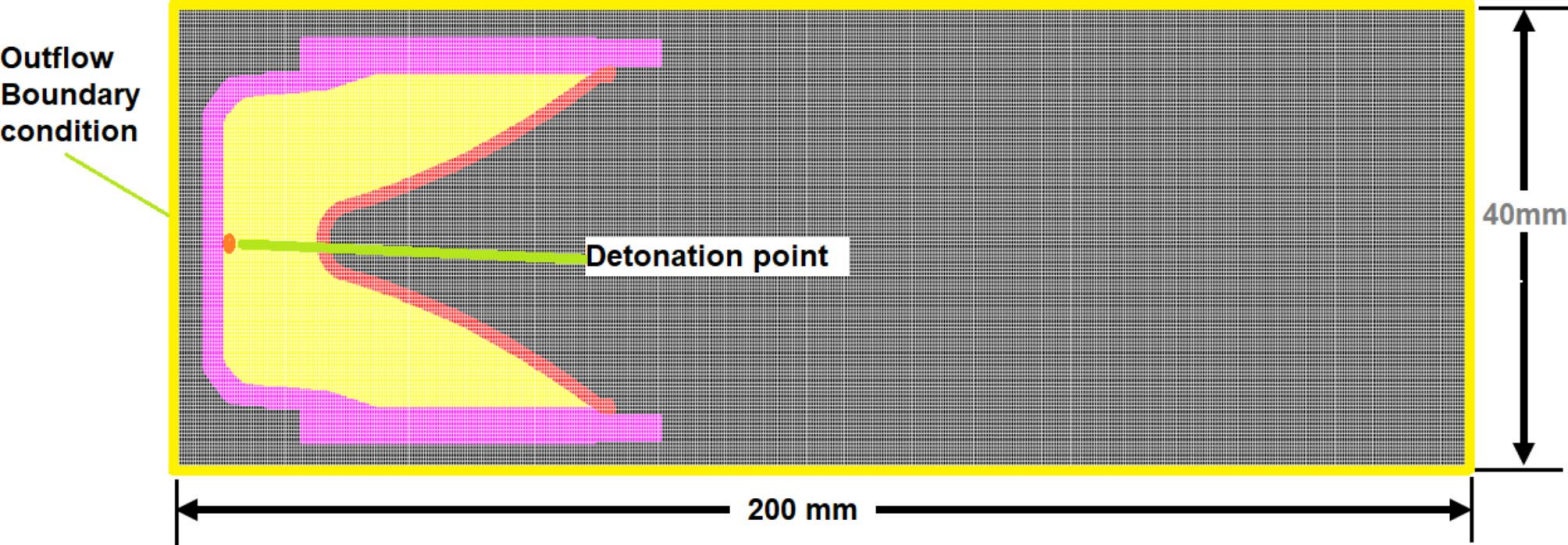
Shaped charge elements within numerical model grids, initial condition and boundary condition.

Four numerical approaches have been implemented within Autodyn as follow:

- The PER theory^32^ based jetting analysis approach, which is implemented to estimate the jet velocities, masses and its kinetic energy and momentum. General square mesh size 0.3 × 0.3 mm cell is used in all jetting analyses for its reasonable accuracy and elapsed time.
- The Euler based jet formation algorithm is used to show the jet profile at different time intervals including the jet temperature, pressure, velocity, etc. Figure 4 shaped charge elements within numerical model grids, initial condition and boundary condition.
- Lagrange method is applied to the jet penetration approach in a steel target, where the jet produced from Euler solver of the jet formation is remapped to another Lagrange grid (with the same mesh size, all related parameters such as element velocity, pressure, density, ….etc), where the penetration depth is determined at different interval time starting from the moment of interaction. Erosion strains of 50% and 600% were used for steel and copper liner materials, respectively, according to Ref.^33^.

More details about the effect of erosion strain and the analysis of the mesh in addition to the verification and validity of the hydrocode are presented in Ref.^33^. The Lagrange mesh size of 0.5 mm×0.5 mm was applied to all penetration simulation calculations considering its reasonable accuracy and time consumption^34^.

d. Smooth particle hydrodynamics (SPH), had proved high efficiency in dealing with the fragmentations problem due to several advantages such as mesh tangling within Lagrangian due to high deformation. Also it offers a model with high efficiency, accurate and fast for the different materials producing fragments. The HTML file of the fragmentation is achieved after finishing the SPH cycles. This file is used to analyse the fragments.

The Euler 2-d shaped charge domain is constructed with length of 200 mm and height of 20 mm, the same cells for both the jet and the target is same one that have been used in Ref.^33^.

### Material model

The models and parameters of the material used within numerical Autodyn will be briefly presented for the explosive and its case, the liner and the material of the target separately.

#### The explosive charge

“Jones Wilkins Lee” (JWL) equation of state was used for the explosive charge^35^, i.e.

1 $$\:p=\text{A}\left(1-\frac{{\upomega\:}}{{\text{r}}_{1}\text{v}}\right){e}^{-{\text{r}}_{1}\text{v}}+B\left(1-\frac{{\upomega\:}}{{\text{r}}_{2}\text{v}}\right){e}^{-{\text{r}}_{2}\text{v}}+\frac{\omega\:E}{\text{v}},$$

where; the symbols A, B, r_1_, r_2_, C and ω are constants^36^, p is the pressure, v is the relative volume (1/ρ) and the specific internal energy per unit mass represented by E. The values of the experimental constants for some explosives have been obtained by the experiments of the sideways plate push dynamic test^37^ and estimated by cylinder expansion test^38–40^. The values of these constants are available in the material library of Autodyn and listed in Table 1 for the studied explosives.

#### The copper liner

The shock model has been applied for the equation of state of the copper liner and its strength model was ignored for high pressure on the liner wall during the collapse of the liner^41^. The experimental tests had proved that the shock velocity (U) values and the material velocity behind the shock (Up) on the Hugoniot of shock might be successfully fitted to a straight line in case of many materials that have not endure a phase change. These values are valid for shock velocities reach to double the initial sound speed C_o_ and the shock pressures in the range of 100 GPa^41^; i.e.

2 $$\:U={C}_{o}+s{U}_{p},$$

where *s* is the material constant which states the relationship of shock velocity-particle velocity by slope.

The Mie-Gruneisen EOS based on the shock Hugoniot is expressed as:

3 $$\:p={p}_{H}+{\Gamma\:}{\uprho\:}(\text{e}-{\text{e}}_{\text{H}}),$$

where Γ is the Gruneisen Gamma coefficient and is equal to *B*_o_/(1+*μ*) where B_o_ is a constant, Γρ = Γ_o_ρ_o_ = constant is assumed; ρ is the density. p_H_ and e_H_ are the Hugoniot pressure and energy, respectively, given by:

**Table 1:** The JWL parameters for the studied explosive charges.

No.	Explosive	Composition	Summary formula	Ref. density (g/cm^3^)	Parameter A (kPa)	Parameter B (kPa)	Parameter r1 (none)	Parameter r2 (none)	Parameter $\:\omega\:$ (none)	Detonation velocity (m/s) [25]	C-J Energy/ unit volume (kJ/m^3^)	C-J Pressure (kPa) [41].
1.	LX-1	Nitromethane 51.7%, tetranitromethane 33.2%, 1-Nitropropane 15.1%	C_1.52_H_3.73_N_1.69_O_3.39_	1.23	3.11 × 10^8^	4.761 × 10^7^	4.50	1.00	0.350	6840	6.10 × 10^6^	1.55 × 10^7^
2.	TNT	TNT	C_7_H_5_N_3_O_6_	1.63	3.738 × 10^8^	3.747 × 10^6^	4.15	0.90	0.350	6930	6.00 × 10^6^	2.10 × 10^7^
3.	PETN 1.5	PETN	C_5_H_8_N_4_O_12_	1.50	6.253 × 10^8^	2.329 × 10^7^	5.25	1.60	0.280	7450	8.56 × 10^6^	2.20 × 10^7^
4.	Pentolite	PETN 50%, TNT 50%	C_2.332_H_2.3659_N_1.293_O_3.2187_	1.70	5.409 × 10^8^	9.373 × 10^6^	4.50	1.10	0.350	7530	8.10 × 10^6^	2.55 × 10^7^
5.	LX-17	92.5%TATB, 7.5% Kel-F	C_2.29_H_2.18_N_2.15_O_2.15_	1.90	4.460 × 10^8^	1.339 × 10^6^	3.85	1.03	0.460	7600	6.90 × 10^7^	3.00 × 10^7^
6.	PBX 9502	95%TATB, 5% Kel-F 800	C_2.3_H_2.23_N_2.21_O_2.21_	1.89	4.613 × 10^8^	9.544 × 10^7^	4.00	1.70	0.480	7710	7.07 × 10^6^	3.02 × 10^7^
7.	TETRYL	Tetryl	C_5_H_5_N_5_O_8_	1.73	5.868 × 10^8^	1.067 × 10^7^	4.40	1.20	0.275	7910	8.20 × 10^6^	2.85 × 10^7^
8.	PBX 9407	RDX 94%, Exon 461 6%	C_1.41_H_2.66_N_2.54_O_2.54_Cl_0.07_F_0.09_	1.60	5.732 × 10^8^	1.464 × 10^7^	4.60	1.40	0.320	7910	8.60 × 10^6^	2.65 × 10^7^
9.	COMP. B	RDX60%, TNT 40%	C_6.851_H_8.75_N_7.65_O_9.3_	1.72	5.242 × 10^8^	7.678 × 10^7^	4.20	1.10	0.340	7980	8.50 × 10^6^	2.95 × 10^7^
10.	Cyclotol	RDX 77%, TNT 23%	C_5.045_H_7.461_N_6.876_O_7.753_	1.75	6.034 × 10^8^	9.924 × 10^7^	4.30	1.10	0.350	8250	9.20 × 10^6^	3.20 × 10^7^
11.	A3	RDX 91%, Wax 9%	C_1.87_H_3.74_N_2.46_O_2.46_	1.65	6.113 × 10^8^	1.065 × 10^7^	4.40	1.20	0.320	8300	8.90 × 10^6^	3.00 × 10^7^
12.	PBX 9010	RDX 90%, Kel-F 10%.	C_1.39_H_2.43_N_2.43_O_2.43_Cl_0.09_F_0.26_	1.79	5.815 × 10^8^	6.801 × 10^6^	4.10	1.00	0.350	8390	9.00 × 10^6^	3.40 × 10^7^
13.	*RDX-V5	RDX 95%, Viton A 5%	C_3.34_H_6.29_N_6_O_5.97_	1.76	6.100 × 10^8^	1.300 × 10^7^	4.50	1.40	0.250	8424	8.78 × 10^6^	3.02 × 10^7^
14.	LX-04	HMX 85%, Viton A 15%	C_5.485_H_9.2229_N_8_O_8_F_1.747_	1.87	8.364 × 10^8^	1.298 × 10^7^	4.62	1.25	0.420	8470	9.50 × 10^6^	3.40 × 10^7^
15.	BTF	Benzotrifuroxane	C_6_N_6_O_6_	1.85	8.407 × 10^8^	1.496 × 10^7^	4.60	1.20	0.300	8480	1.15 × 10^7^	3.60 × 10^7^
16.	OCTOL	HMX 76.3%, TNT 23.7%	C_6.835_H_10.025_N_9.215_O_10.43_	1.82	7.490 × 10^8^	1.340 × 10^7^	4.50	1.20	0.380	8480	9.60 × 10^6^	3.42 × 10^7^
17.	PBX 9011	HMX 90%, Estane 10%	C_5.696_H_10.476_N_8.062_O_8.589_	1.78	6.347 × 10^8^	7.998 × 10^6^	4.20	1.00	0.300	8500	8.90 × 10^6^	3.40 × 10^7^
18.	*BCHMX-V5	BCHMX 95%, Viton A 5%	C_4.45_H_6.41_N_8_O_7.99_	1.81	3.970 × 10^8^	1.710 × 10^7^	4.07	0.98	0.374	8612	1.01 × 10^6^	3.30 × 10^7^
19.	LX-07	HMX 90%, Viton A 10%	C_1.48_H_2.62_N_2.43_O_2.43_	1.87	8.710 × 10^8^	1.389 × 10^7^	4.60	1.15	0.300	8640	1.00 × 10^7^	3.55 × 10^7^
20.	*HMX-V5	HMX 95%, Viton A 5%	C_4.42_H_8.43_N_8_O_8.03_	1.84	7.780 × 10^8^	7.070 × 10^7^	4.20	1.00	0.300	8730	9.18 × 10^6^	3.35 × 10^7^
21.	LX-14	HMX 95.5%, Estane 4.5%	C_4.8_H_9.1365_N_8.024_O_8.2811_	1.84	8.261 × 10^8^	1.724 × 10^7^	4.55	1.32	0.380	8800	1.02 × 10^7^	3.70 × 10^7^
22.	PBX 9404	HMX94%, NC (12%N) 3%, Tris(β-chloroethyl) phosphate 3%	C_4.42_H_8.659_N_8.075_O_8.4754_Cl_0.0993_ P_0.033_	1.84	8.524 × 10^8^	1.802 × 10^7^	4.60	1.30	0.380	8800	1.02 × 10^7^	3.70 × 10^7^
23.	PBX 9501	HMX 95%, Estane 2.5%, BDNPA, 1.25%, BDNPF 1.25%	C_1.47_H_2.86_N_2.6_O_2.69_	1.84	8.524 × 10^8^	1.802 × 10^7^	4.55	1.30	0.380	8800	1.02 × 10^7^	3.70 × 10^7^
24.	LX-10	HMX 95%, Viton A 5%	C_1.425_H_2.6831_N_2.566_O_2.566_	1.87	8.807 × 10^8^	1.836 × 10^7^	4.62	1.32	0.380	8820	1.04 × 10^7^	3.75 × 10^7^
25.	HMX	HMX	C_4_H_8_N_8_O_8_	1.89	7.783 × 10^8^	7.071 × 10^6^	4.20	1.00	0.300	9110	1.05 × 10^7^	4.20 × 10^7^
26.	*CL-20-V5	CL-20 95%, Viton A 5%	C_6.69_H_6.5_N_12_O_11.94_	1.95	1.640 × 10^9^	1.860 × 10^8^	6.50	2.70	0.550	9194	9.95 × 10^6^	3.81 × 10^7^

*The JWL parameters of the BCHMX explosive were calculated from the Mat Lab Simulink program based on intensive calculations considering various densities and relevant detonation pressure values. These values were obtained from the EXPLO5 code at different densities leading to different explosive characteristics, and hence optimized JWL equation of state parameters were estimated analytically.

4 $${p_H}=\frac{{{\rho _o}c_{o}^{2}\mu \left( {1+\mu } \right)}}{{{{\left[ {1 - \left( {s - 1} \right)\mu } \right]}^2}}}$$

and

5 $${e_H}=\frac{1}{2}\frac{{{p_H}}}{{{\rho _o}}}\left( {\frac{\mu }{{1+\mu }}} \right),$$

where µ=(ρ/ρ_o_)-1 represents the compress-ability. Table 2 presents the mechanical characteristics of the copper liner, where the constants of the previous equations were obtained from the materials library.

#### The charge case

The charge case material is steel 1006 with shock EOS that had been depicted for the material of the liner, while the strength model was ignored. The shock EOS parameters for the casing material of the charges are described in Table 2.

#### Rolled homogeneous armour (RHA) target

The equation of state of the RHA target material is shock with neglecting its strength model. The shock EOS parameters of the target materials were discussed in Sect. 3.1.2, where the different parameters of the shock equation of state are listed in Table 2.

**Table 2:** The mechanical properties of liner, the casing and the RHA target materials^25^.

Parameter	Copper	Steel 1006 ethylene	RHA target
Equation of state	Shock	Shock	Shock
Reference density (g/cm^3^)	8.93	7.89	7.86
Gruneisen Coefficient	2.02	2.17	1.67
Parameter C (m/s)	3940	4569	4610
Parameter S (non)	1.489	1.49	1.73
Ref. temperature (K)	300	300	300

## Results

### The shaped charges jet characteristics

**Fig. 5:**
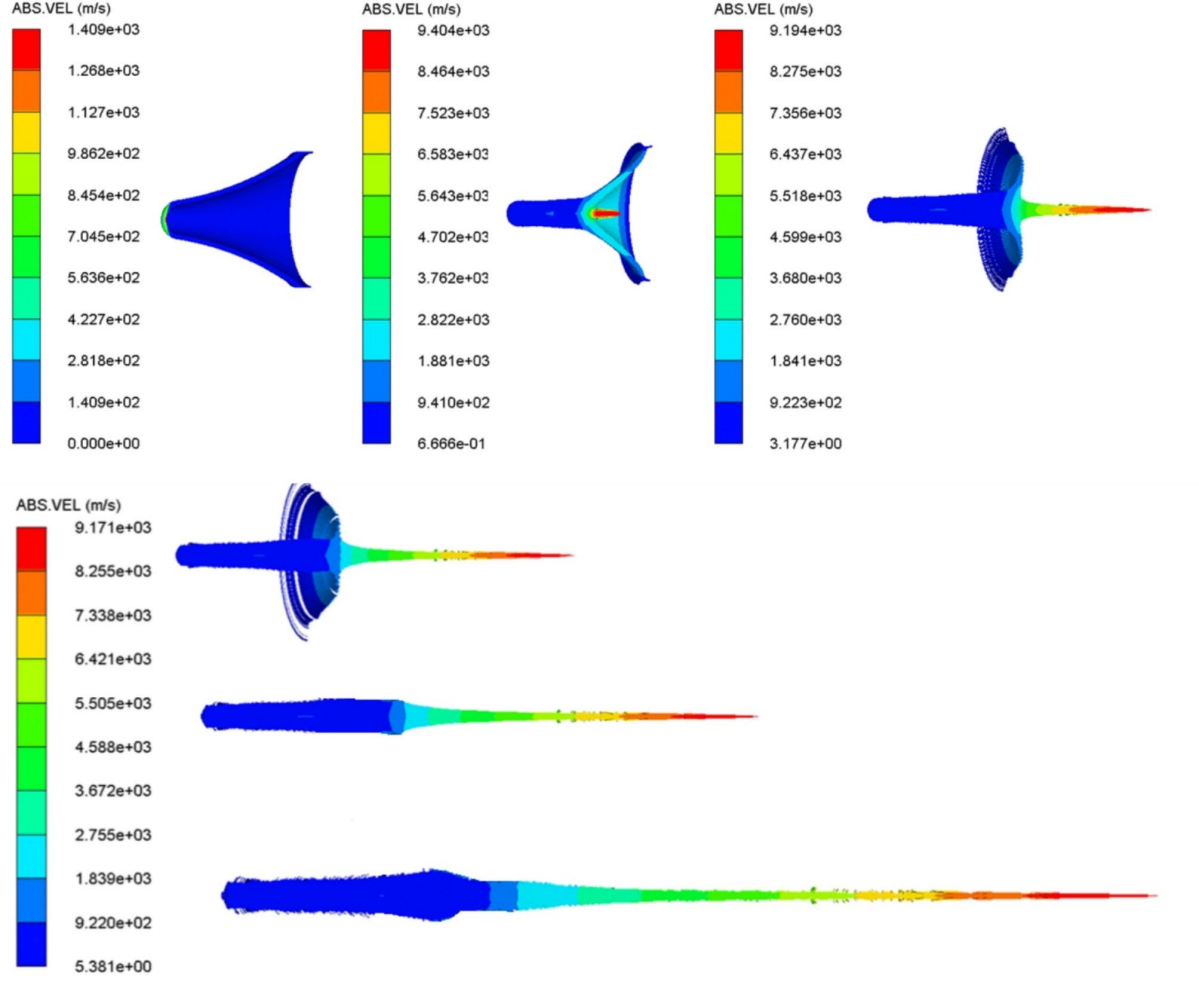
The HMX-V5 shaped charge liner collapse with the jet stretching at different interval times starting from the detonation moment at 1, 7, 12, 16, 19, 24µs respectively.

The numerical models of different shaped charges loaded by various explosive loads are allowed to detonate until the numerical solution that represents the jet formation during its stretching is finished. Figure 5 represents the numerical jet formation algorithm for the loaded HMX-V5 explosive. The starting of the jet formation begins at 7µs, after which the jet length increases and its mass accumulate until the moment of breakup. The scale of the absolute velocity of the jet is observed not to be constant due to the variation between the collapse and the jet velocity at early stages of jet formation generally before 16 µs, after which the steady state jet tip velocity of 9171 m/s is sustained.

**Fig. 6:**
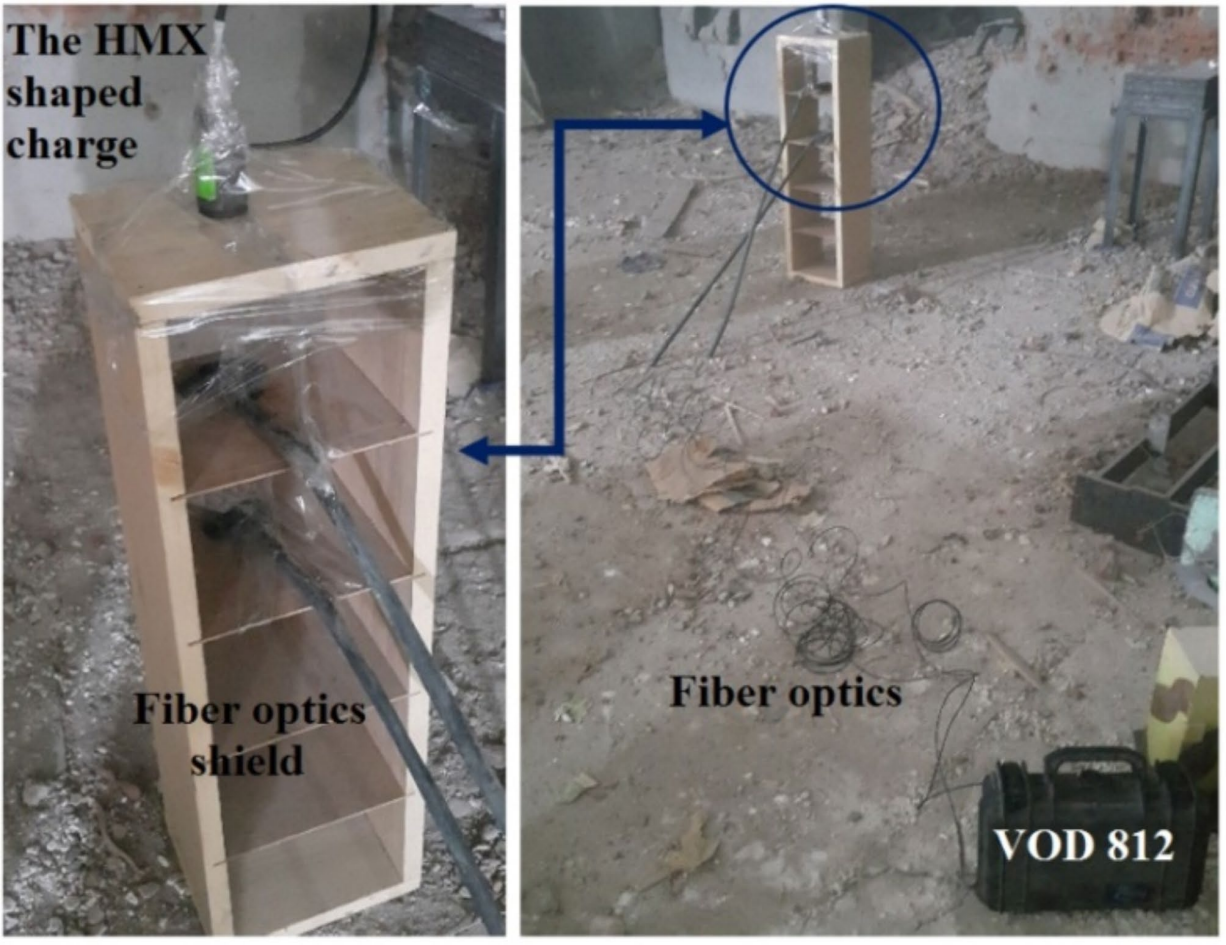
The test setup applied to measure the jet tip velocity of the HMX-V5 charge using vod-812 apparatus by OZM research.

In order to validate the numerical shaped charge jetting analysis that includes the jet tip velocity, an experimental shaped charge filled with HMX-V5 explosive has been fired statically. It generates hypervelocity jet, which was allowed to penetrate through two wooden frames spaced by 15 cm. Each wooden frame has an attached fibre optic probe of the OZM VOD 812 apparatus attached to its upper surface to show the start-end time of the jet passing through them as shown in Fig. 6. By measuring the distance between the spaced fibre optics and the time difference between the start and end arrival times, the jet tip velocity was calculated precisely. The experimental jet tip velocity had a measured value of 9020 m/s compared to 9354 m/s of the calculated one, which means an error less than 3.6%. This result confirms the velocity of the hypervelocity jet and also validates the used numerical Autodyn hydrocode.

After the validation of the jet tip velocity calculated by the used Autodyn hydrocode, the shaped charge jetting analysis algorithms was applied to the other 25 shaped charges filled with these explosive types. The elemental jet velocities and relevant masses are collected for further analysis.

**Fig. 7:**
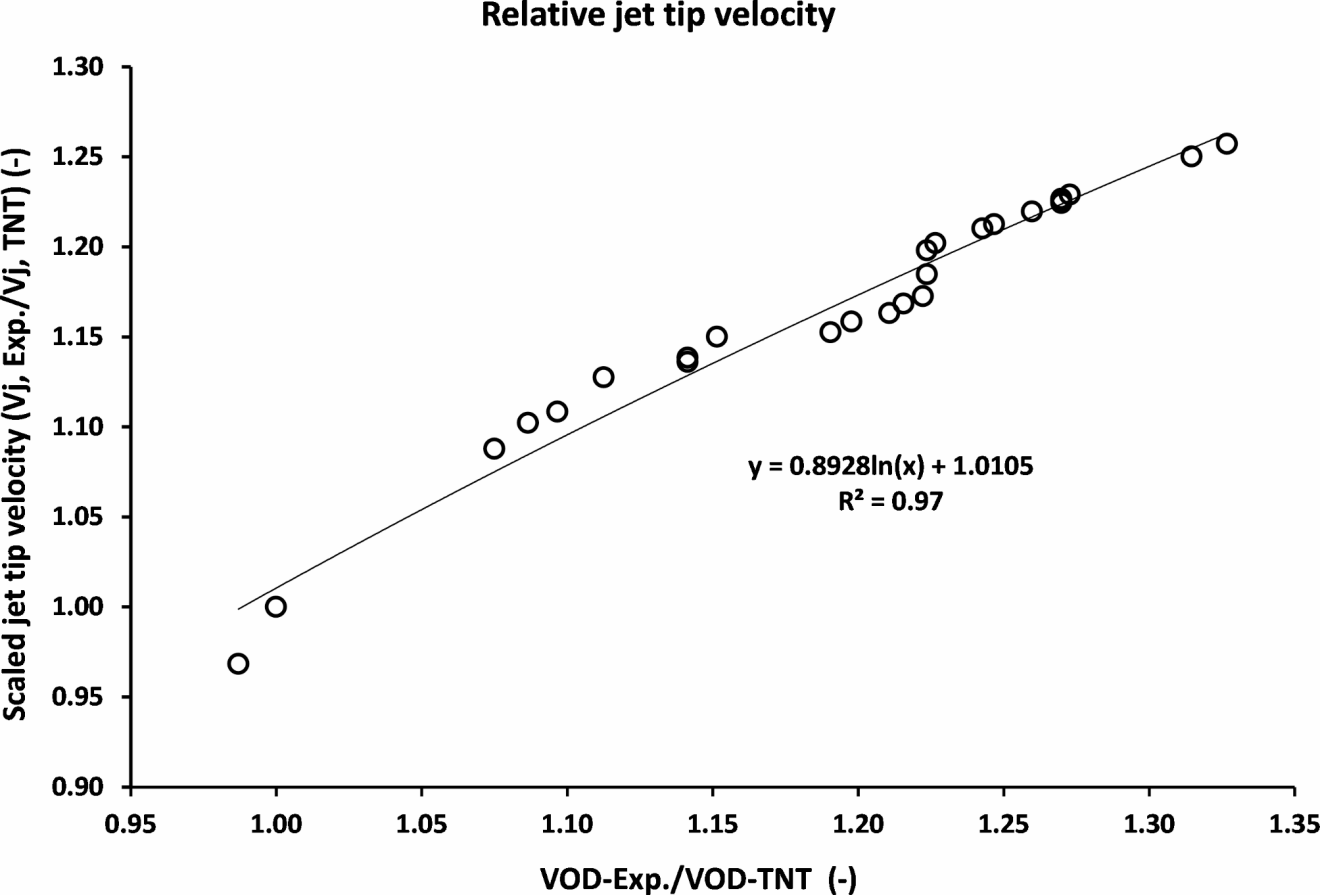
The reliance of the jet tip velocity on the scaled detonation velocity relative to that of the TNT baseline.

Figure 7 shows the relation between the scaled jet tip velocities of the studied explosives (jet tip velocity using different explosives divided by that of the TNT) with the scaled detonation velocity relative to that of the TNT. Explosive LX-1 shows the minimum jet tip velocity of 7410 m/s due to its lowest density of 1.23 g/cm^3^, and thus its lowest detonation velocity of 6840 m/s. while the explosive CL20-V5 exhibited the highest jet tip velocity of 9621 m/s, which was attributed to its largest detonation velocity and thus the collapse velocity of the liner element due to the high performance of CL-20 that was found to be the largest among the studied explosive types.

**Fig. 8:**
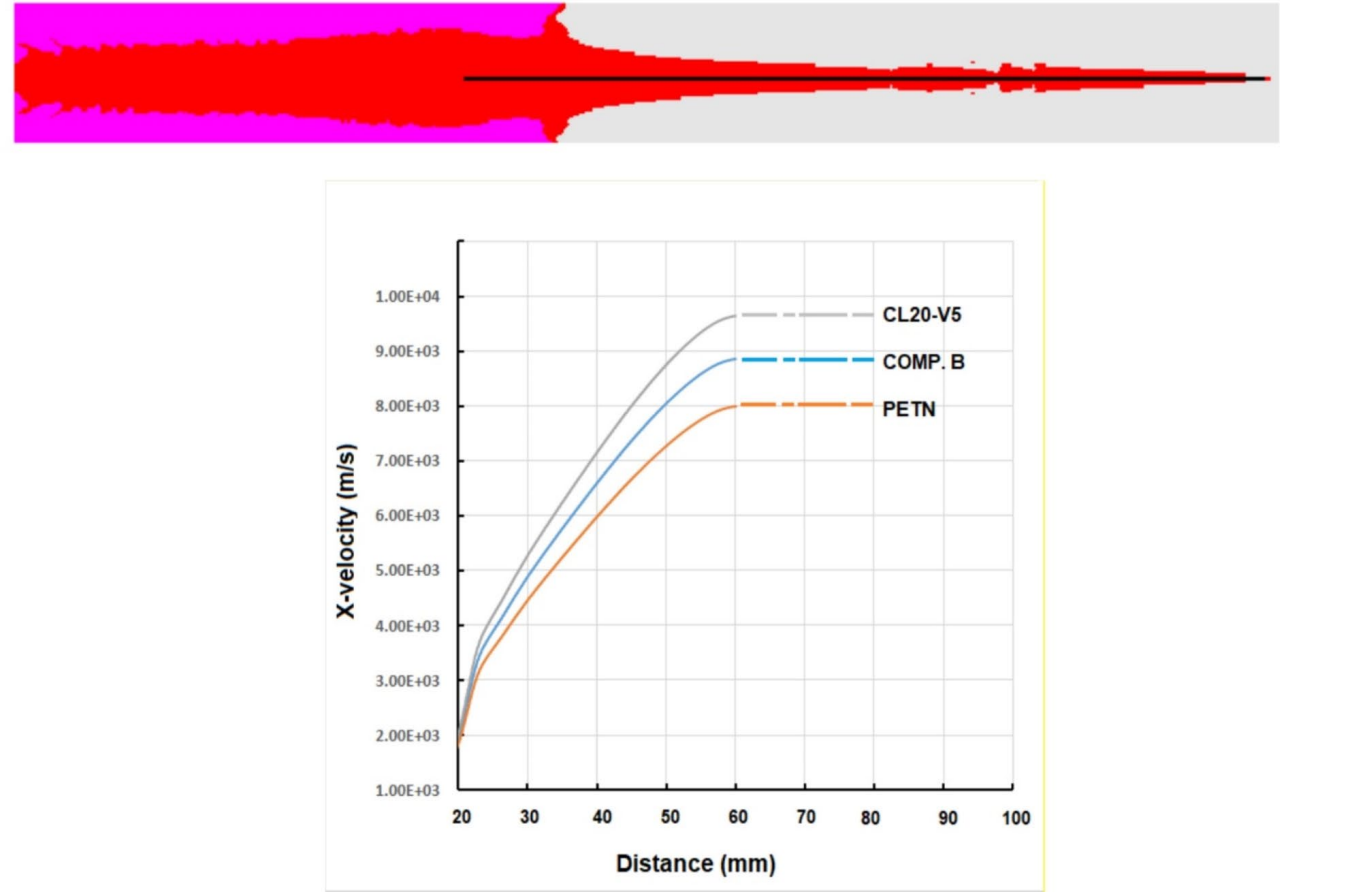
Velocity-distance profile for shaped charge jet using different explosives using Euler jet formation solver.

To compare the profile of velocity-distance for the studied explosives, only three velocity profiles for lower, medium and high velocity of detonation cases have been selected for comparison. This can be adapted here in Fig. 8. The explosive CL20-V5, which exhibits the highest detonation velocity of 9.1 km/s shows the greatest jet tip velocity of 9.6 km/s, other than PETN having the lowest tip velocity of 8 km/s. Initial jet length before stretching for the three cases is about 35 mm, after which every jet begins to elongate until its particulation.

**Fig. 9:**
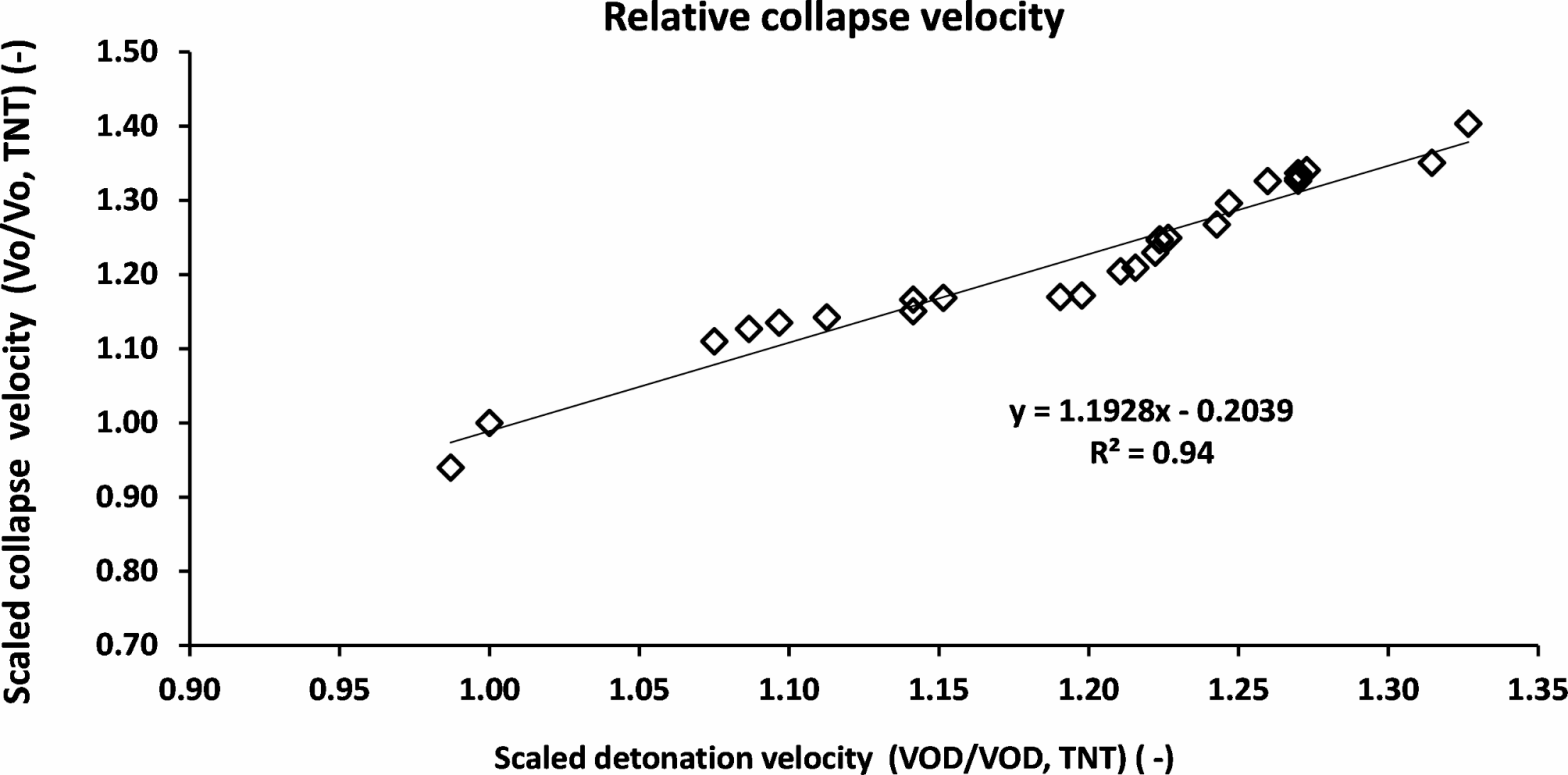
The scaled collapse velocity relation with scaled detonation velocity relative to TNT explosive material.

Figure 9 shows the relation between the scaled collapse velocities with the scaled detonation velocity with respect to TNT as a baseline. The scaled collapse velocity of the CL20-V5 explosive was found to be 140% of the TNT explosive, which in turn gives a relevant jet tip velocity of 26% greater than that of that of the TNT explosive.

Based on data fitting of Fig. 7, the relative jet tip velocity can be estimated via:

6 $$\:{\frac{V{\:}_{j,\:expl}}{V{\:}_{j,TNT}}\:=0.8928\text{ln}\:(\frac{VOD,\:{\:}_{expl}}{6930})+1.0105\:.}_{\:}$$

For HMX-V5 explosive, VOD = 8730 m/s; the jet tip velocity can be approximated as:

Vj/Vj, TNT = 1.2166.

The jet tip velocity of TNT explosive charge = 7653 m/s; thus the calculated jet tip velocity of the HMX-V5 explosive charge using the empirical equation = 9310 m/s. This value is only 3.22% different from that of the measured jet tip velocity of 9020 m/s.

### Jet mass and relevant kinetic energy

**Fig. 10:**
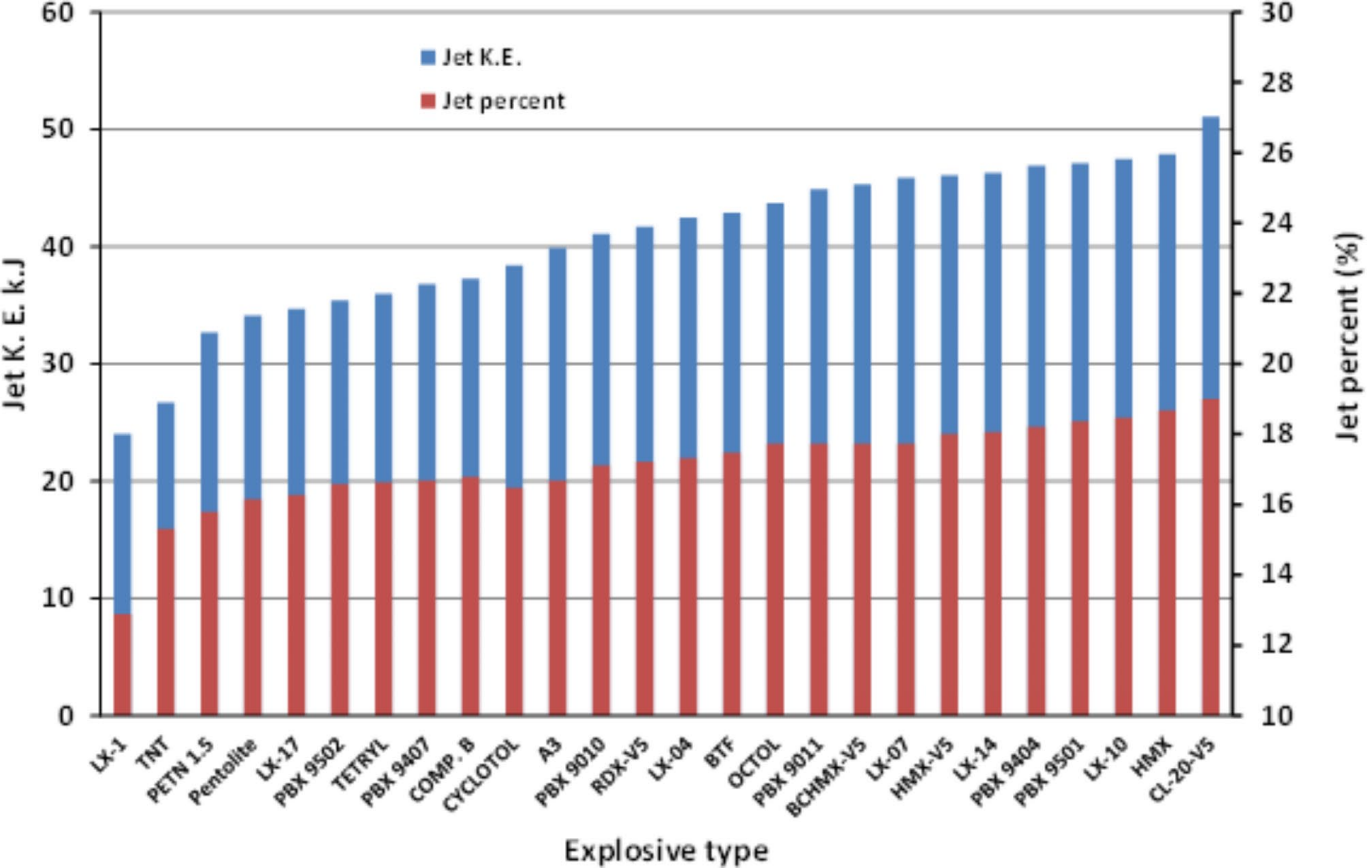
The calculated jet mass percentages and the relevant kinetic energy of the shaped charge jets loaded with various explosive materials.

Figure 10 summarizes the calculated jet mass percentages and the relevant kinetic energy of the shaped charge jets loaded with various explosive materials. The mass of the jet was obviously increases as the detonation velocity of the used explosive increases. The TNT shaped charge jet has kinetic energy of 26.7 kJ, whereas the kinetic energy of the CL20-V5 shaped charge jet is 51.1 kJ. The reason behind this was explained in the elemental jetting analysis including the collapse velocity and its relevant elemental jet velocity. The total jet kinetic energy was found to be a measure and an evidence for the shaped charge jet penetration potential as was discussed by Davinson and Pratt^11^, where the penetration depth into concrete was enhanced by 28% as the jet kinetic energy increased by 10%. This increase in the cumulative kinetic energy of the CL20-V5 jet (about 200% in comparison with the baseline TNT explosive material (i.e. 24 kJ for TNT and 51.1 kJ for CL20-V5)) is expected to have significant influence on the depth of penetration when these charges are fired against the same RHA targets.

The variation in the jet velocities and the jet kinetic energy of these shaped charges loaded with various explosives is attributed to the different Gurney energy values or Gurney velocity delivered to the liner element. The Gurney velocity of this explosive, which is a characteristic property of a certain explosive, represents the energy liberated from the high explosive, which is delivered to the liner element and therefore transformed into mechanical work. The Gurney velocity had increased by increasing the explosive detonation velocity and simultaneously its detonation pressure, which resulted in increasing the jet tip velocity.

On the same time, the jet kinetic energy and its penetration ability into the target will be increased. Elshenawy et al.^42^ related the Gurney velocity to an empirical equation including C-J pressure, explosive loading density and its specific impulse values. Also, the Gurney velocity is related to the detonation velocity of the used explosive charge load as per ref.^43^.

### Proposed analytical model for liner collapse

**Fig. 11:**
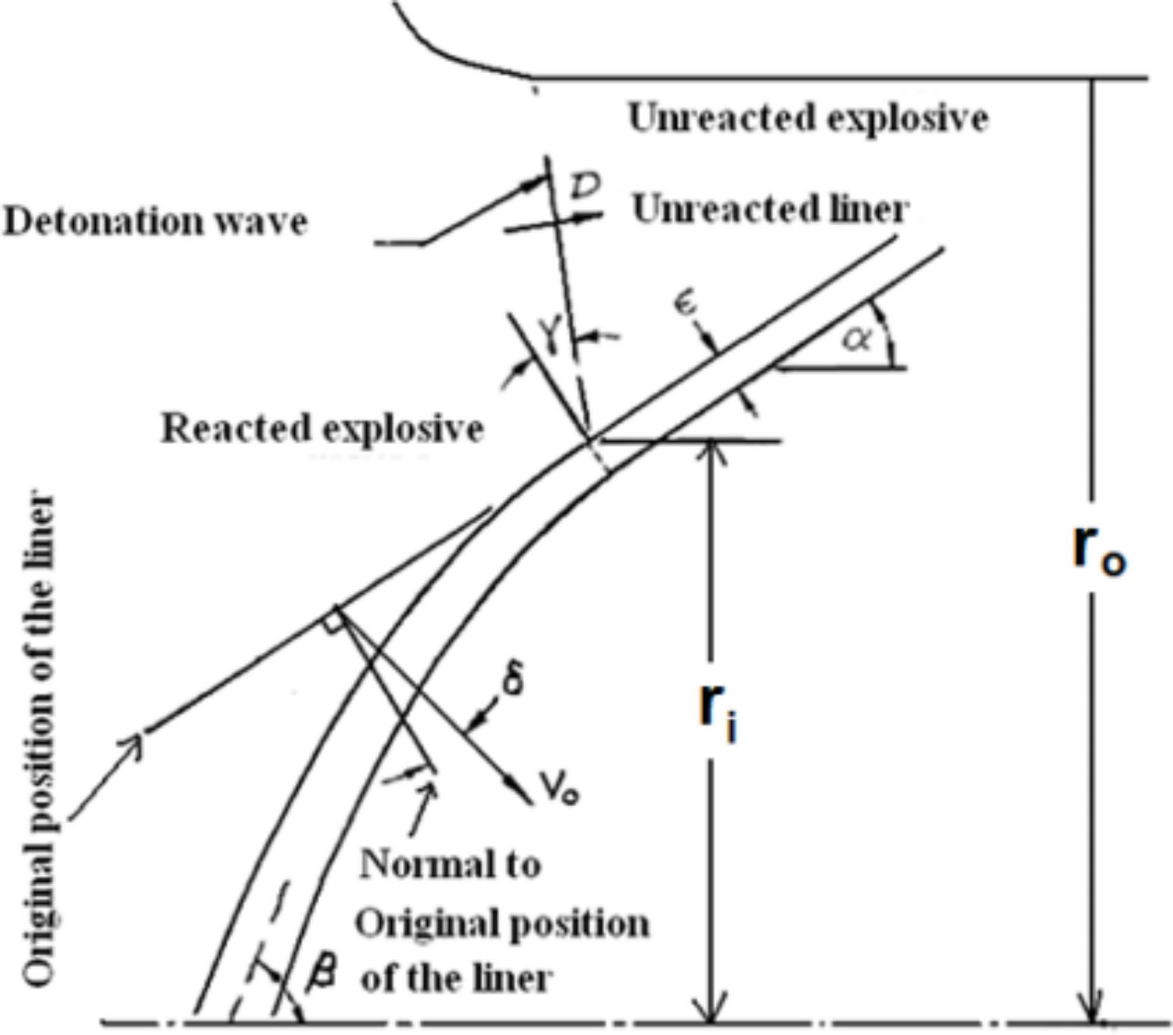
A schematic diagram of the collapsing liner under explosive load.

Using the same concept of imploding cylinder charges as this of Kleinhanss^44^, depending on the same governing parameters that have direct influence on the liner velocity on certain parameters such as liner and explosive diameters, liner thickness and explosive detonation velocity, semi-empirical relation has been proposed. To do this, the collapse velocity obtained numerically for the entire explosives, has been used to obtain such semi-empirical relation, which approximates the collapse velocity depending on the presented influential parameters according to the Kleinhanss model. It was observed experimentally that the liner collapse velocity depends upon the radius of the cylinder explosive charges, i.e.

7 $$\:{V}_{o}={U}_{D}\left[\frac{{r}_{i}-\sqrt{\epsilon\:\left(2{r}_{i}-\epsilon\:\right)}}{{r}_{i}-\epsilon\:}\:.\frac{1}{\left[{C}_{o}+\epsilon\:f\left(b\right)\right]}\right]\:,\:\:\:\:\:$$

where ε is the metal liner thickness, b = r_o_-r_i_ is the explosive thickness, r_o_ and r_i_ are the outer and inner explosive radii, respectively. C_o_ and *f*(b) are empirical parameters that depend on the used explosive and metal liner. These parameters are shown in schematic diagram; Fig. 11.

Thus,

8 $$\:\raisebox{1ex}{$\left(\:\frac{{V}_{o}}{D}\right)$}\!\left/\:\!\raisebox{-1ex}{$\left[\frac{{r}_{i}-\sqrt{\left({2r}_{i}-\right)}}{{r}_{i}-}\right]$}\right.=\frac{1}{[{C}_{o}+f(b\left)\right]}.$$

**Fig. 12:**
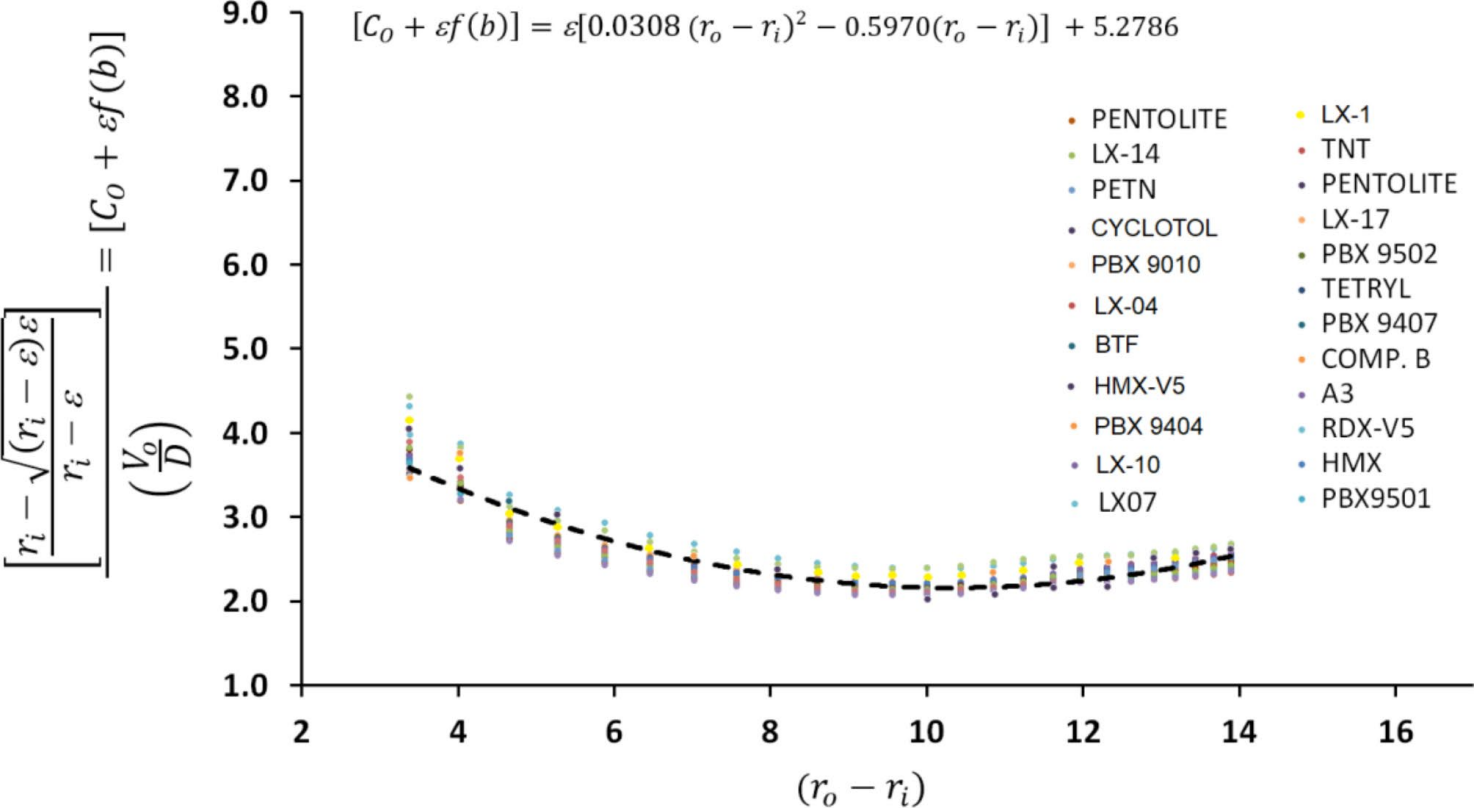
The interpolation used to estimate the collapse velocity with respect to the detonation velocity and the liner and explosive thicknesses.

Therefore, the non-dimensional number in Eq. (8) has been used with the explosive thickness to estimate the empirical constants (i.e. RHS of Eq. (8)) as depicted in Fig. 12 using:

9 $$\:{\left[{C}_{o}+f\left(b\right)\right]=[a}_{\:}({{r}_{o}-{r}_{i})}^{2}-b({{r}_{o}-{r}_{i}\left)\right]}^{\:}+c.$$

Thus, the empirical constants; a, b, c have been obtained using the best fit of every explosive alone, then an average value have been obtained as follow:

10 $$\:{\left[{C}_{o}+f\left(b\right)\right]=[0.0308}_{\:}({{r}_{o}-{r}_{i})}^{2}-0.5970({{r}_{o}-{r}_{i}\left)\right]}^{\:}+5.2786.$$

The general collapse velocity can then be approximated using the following approximation:

11 $$\:\:\left(\:\frac{{V}_{o}}{D}\right)=\left[\frac{{r}_{i}-\sqrt{\left(2{r}_{i}-\right)}}{{r}_{i}-}\right].\frac{1}{{[0.0308}_{\:}({{r}_{o}-{r}_{i})}^{2}-0.5970({{r}_{o}-{r}_{i}\left)\right]\:}^{\:}+5.2786}.$$

To confirm the validity of the proposed formulae; the maximum obtained error between the estimated collapse velocity based on the proposed model; Eq. (11), and that obtained by the standard jetting analysis is listed in Table 3 for the global comparison. It can be concluded that the maximum error was recorded for the CL20-V5 explosive of 4.57%.

**Table 3:** The calculated jetting collapse velocity the obtained one using eq. (11) and the maximum error percent.

Explosive	V_o_ calc. Eq. (10) (m/s)	V_o_ jetting (m/s)	Max. error (%)	Explosive	V_o_ calc. Eq. (10) (m/s)	V_o_ jetting (m/s)	Max. error (%)
LX-1	1316	1300	1.26	LX-04	1630	1577	3.36
TNT	1334	1320	1.03	BTF	1632	1600	2.00
PETN 1.5	1434	1481	-3.19	Octol	1632	1604	1.74
Pentolite	1449	1486	-2.48	PBX 9011	1636	1577	3.73
LX-17	1463	1412	3.58	BCHMX-V5	1657	1590	4.24
PBX 9502	1484	1434	3.47	LX-07	1663	1599	3.99
Tetryl	1522	1471	3.48	HMX-V5	1680	1620	3.71
PBX 9407	1522	1493	1.96	LX-14	1694	1630	3.90
Comp. B	1536	1525	0.70	PBX 9404	1694	1642	3.14
Cyclotol	1588	1530	3.77	PBX 9501	1694	1730	-2.11
A3	1597	1544	3.45	LX-10	1697	1650	2.87
PBX 9010	1615	1580	2.19	HMX	1753	1830	-4.20
RDX-V5	1621	1590	1.96	CL-20-V5	1769	1692	4.57

### Flash x-ray radiograph

**Fig. 13:**
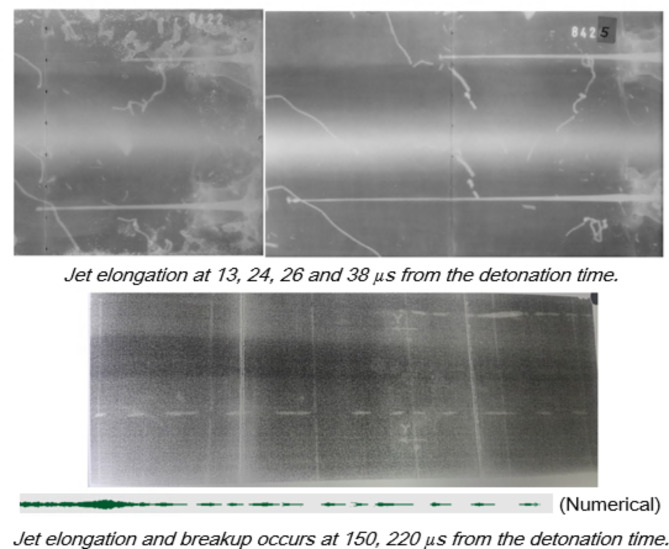
Jet profiles x-ray image at different times from the detonation moment.

The initial delay times at which the x-ray trial photos have been set were. 13, 24, 26, 38µs for continuous jet as shown in Fig. 13; whereas particulation times of 150 and 220µs have been assigned for complete profile at broken up jet elements as depicted in the same figure.

The jet tip velocity has 2.1% different from that of the numerical simulation. (Average V_tip_-x-ray = 9160 m/s). Unfortunately we couldn’t have more than few verification measurements of the jet tip velocity due to its high cost.

**Fig. 14:**
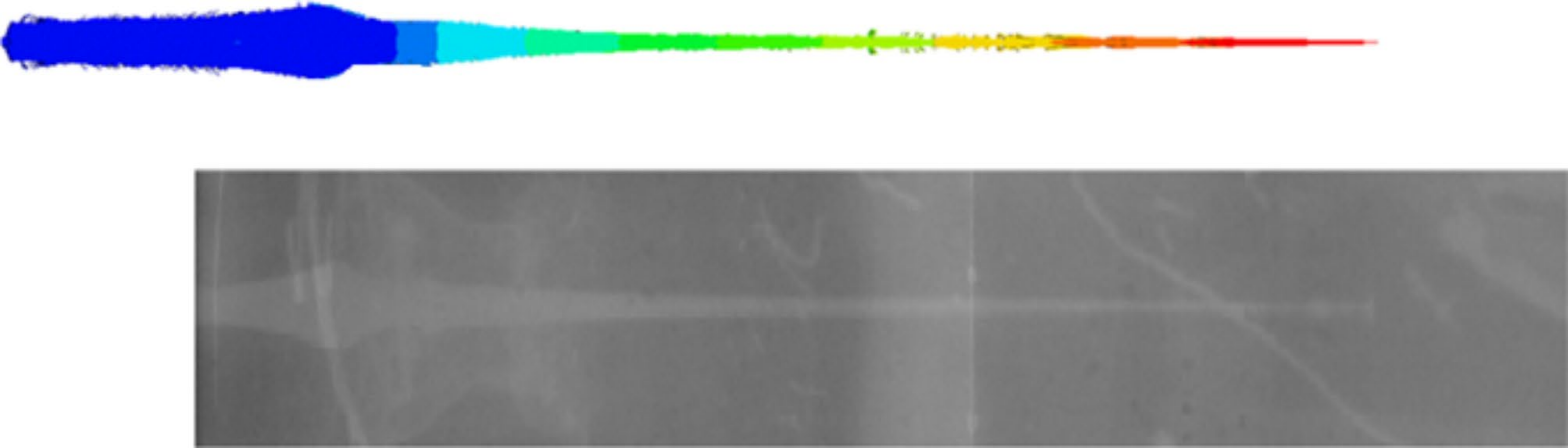
Comparison of jet profiles (numerical and x-ray trial) nearly at the same time.

In accordance with obtained jet profile at different times before particulation and breaking up, the jet symmetry shows complete alignment of liner and explosive elements and also confirm the ductility of the evolved jet especially at stand-off distances lower than 6 times the charge diameter. Besides, Fig. 14 compares between the two jet profiles at 25 µs of both the numerical and the obtained flash x-ray radiograph nearly at the same time, which confirms the jet length and validates and also verifies the used Autodyn hydrocode.

### Fragmentation calculation analysis for different explosives

The numerical SPH fragmentation will not be validated within our research; but the same steps used for the 120 mm shells^45^ have been followed using the same SPH particle number and the same methodology for the filled explosives for the calculated fragmentation analysis using different explosive loads for the studied shaped charges. The used packing size for the entire 26 explosive charge fragmentation casing was 0.3, which shows an affordable time consumption of **80 h** (time step 5 × 10^− 8^ ns (nano-second)), below which the computations cannot proceed anymore and the solution become divergent. (Packing effect parameters are listed in Table 4).

**Table 4:** SPH packing, computation time and relevant time step for fragmentation models.

SPH size	0.1	0.3	0.5	0.7	0.9	1.5	2
Nodes	700,000	302,521	64,054	21,520	7214	3680	1660
Computation time (hr)	250	80	50	19	9	5	2
Time step (ns)	3 × 10^− 11^	5 × 10^− 8^	3.5 × 10^− 7^	8.7 × 10^− 6^	4.5 × 10^− 6^	3.1 × 10^− 5^	7 × 10^− 5^

**Fig. 15:**
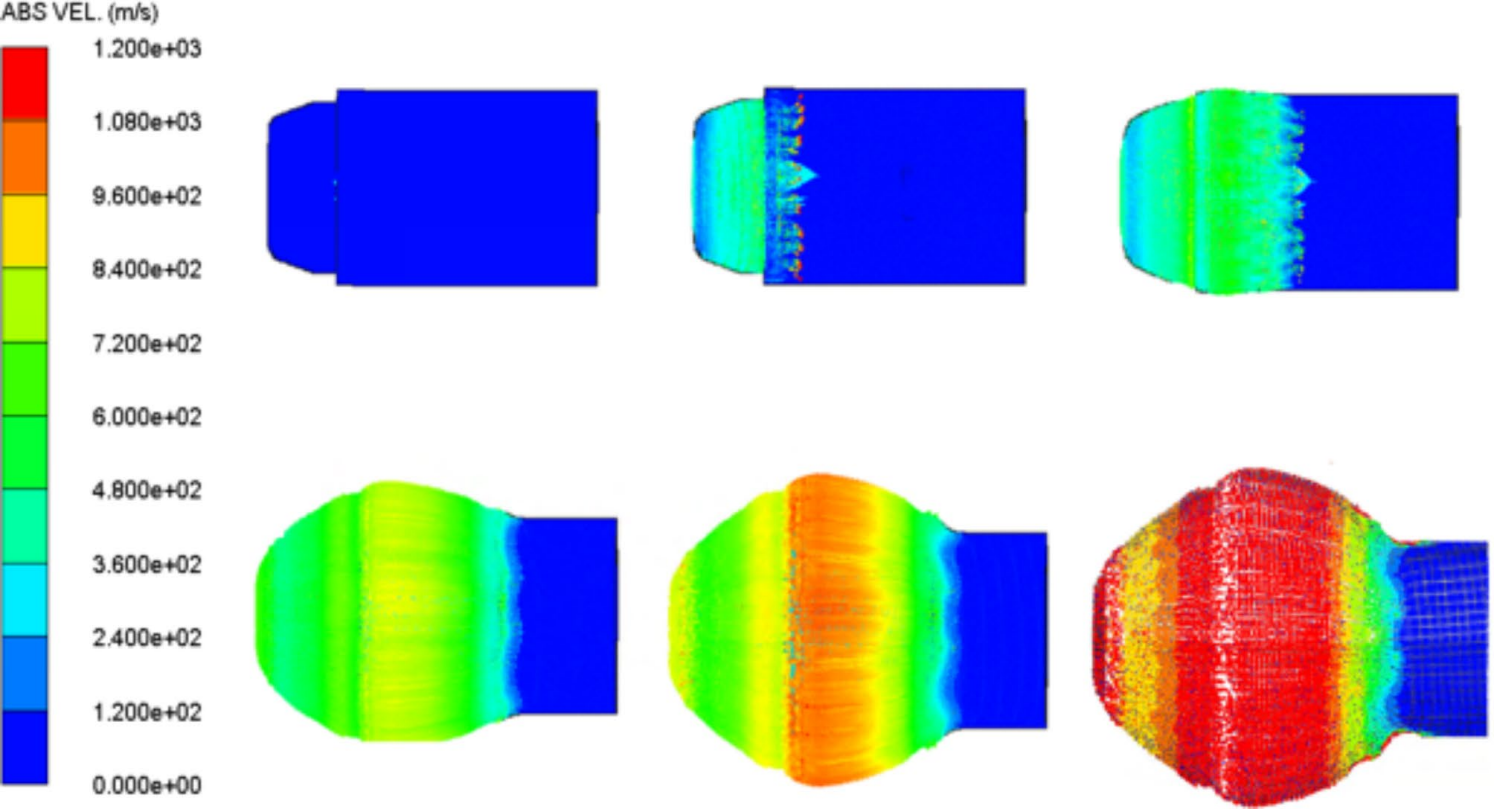
The SPH fragmentation pattern of a tested shaped charge at 0, 2.7, 4.2, 9, 11.9, and 14.83 µs from the moment of detonation.

Sample of the fragmentation pattern using SPH technique to the studied shaped charge is shown in Fig. 15 at different times.

**Fig. 16:**
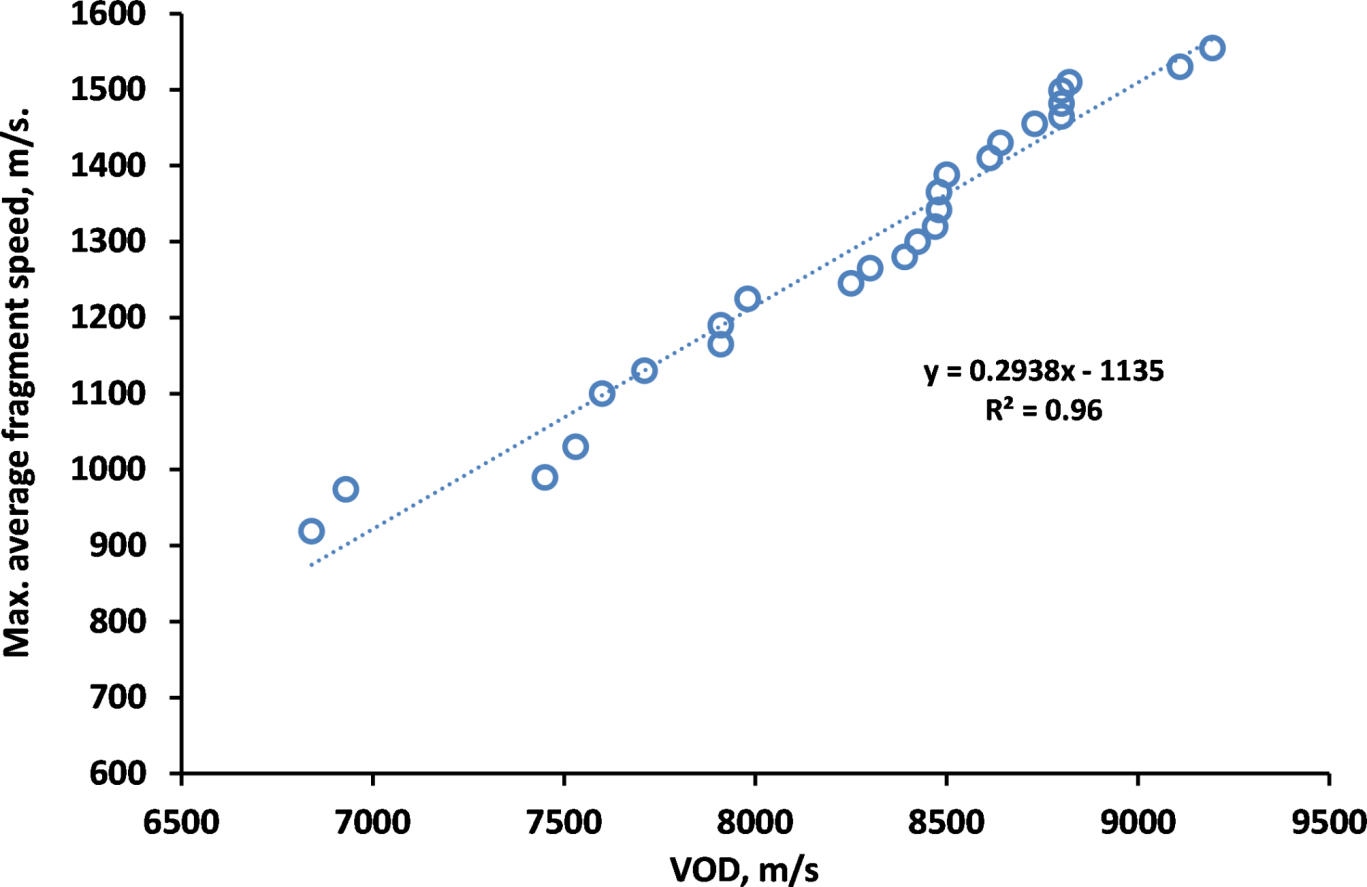
A relationship between the detonation velocities and the average maximum fragments velocities.

The Gurney velocity with different characteristic of each explosive produces different fragments velocities, whereas the internal energy of the explosives presented a large difference in the number of fragment. Also Fig. 16 shows a linear relation between detonation velocity of the used explosives and maximum average fragment speed for the studied explosives. LX-1 explosive had the minimum value of detonation velocity and produced the lowest average fragments’ velocity about 920 m/s; whereas the CL20-V5 produced the largest velocity of fragments with value more than 1550 m/s and also showed the lowest mass of fragments.

**Fig. 17:**
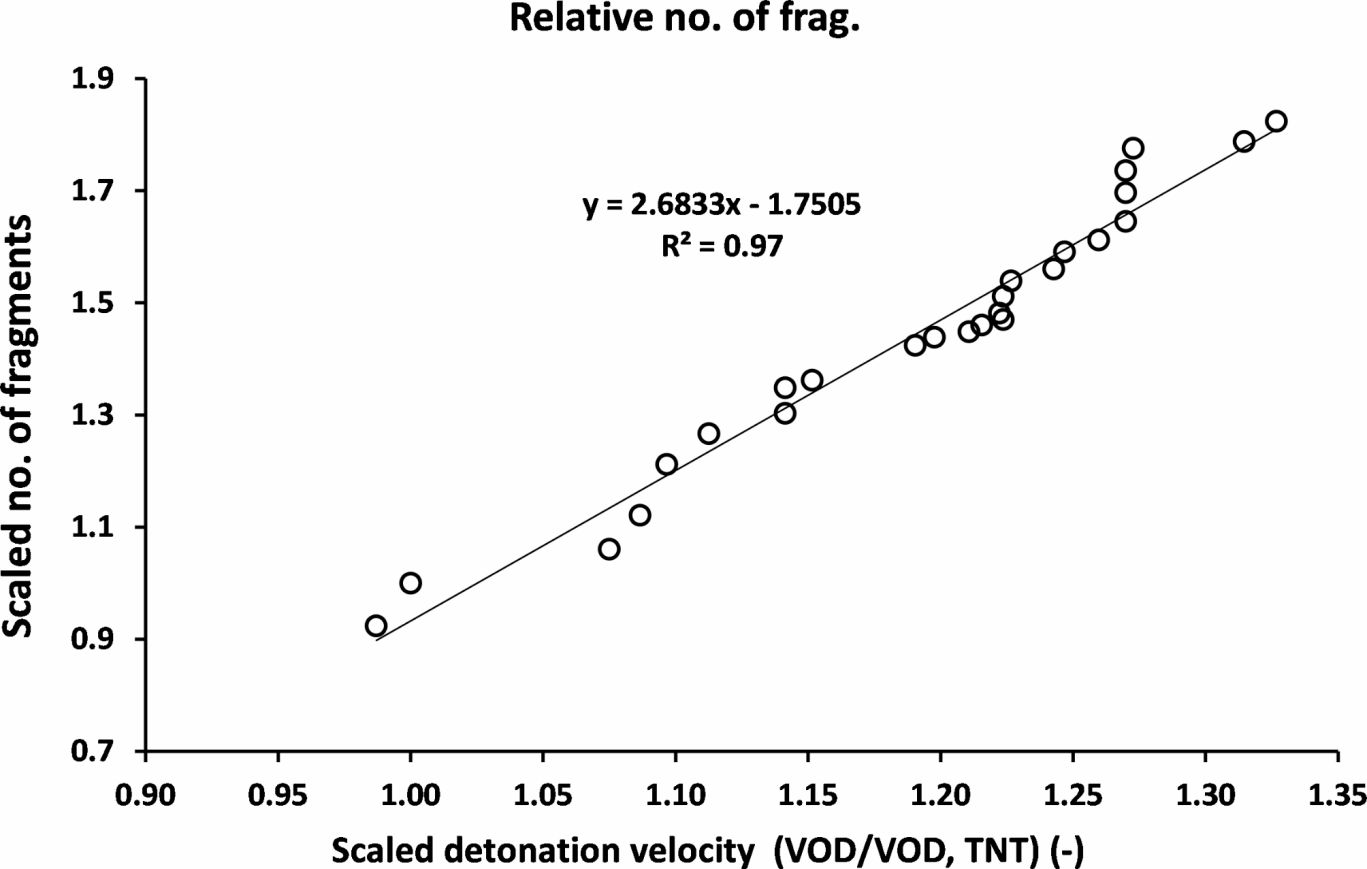
The relation between the scaled detonation velocity and the scaled number of fragments.

Zhou et al.^46^ have studied different types of explosives and their fragmentation ability based on the TNT equivalent. In this study, a relationship between the scaled detonation velocity and the scaled number of fragments is presented in Fig. 17. The number of fragments obtained from the CL20-V5 explosive was found to be more than the baseline TNT explosive material by 1.82 times. Whereas the lowest number of fragments was achieved with the least efficiency LX-1 explosive material, which has 0.92 times the number of fragments of the TNT explosive. It is clear from the results that the number of fragments increased by increasing the detonation velocity of the tested explosive.

The average fragment speed has been estimated using the high speed camera when the static firing has been performed. The estimated average fragment speed of the HMX-V5 explosive was measured by 1495 m/s in comparison with the calculated value of 1530 m/s, which means an error of 2.3%.

### Penetration testing

**Fig. 18:**
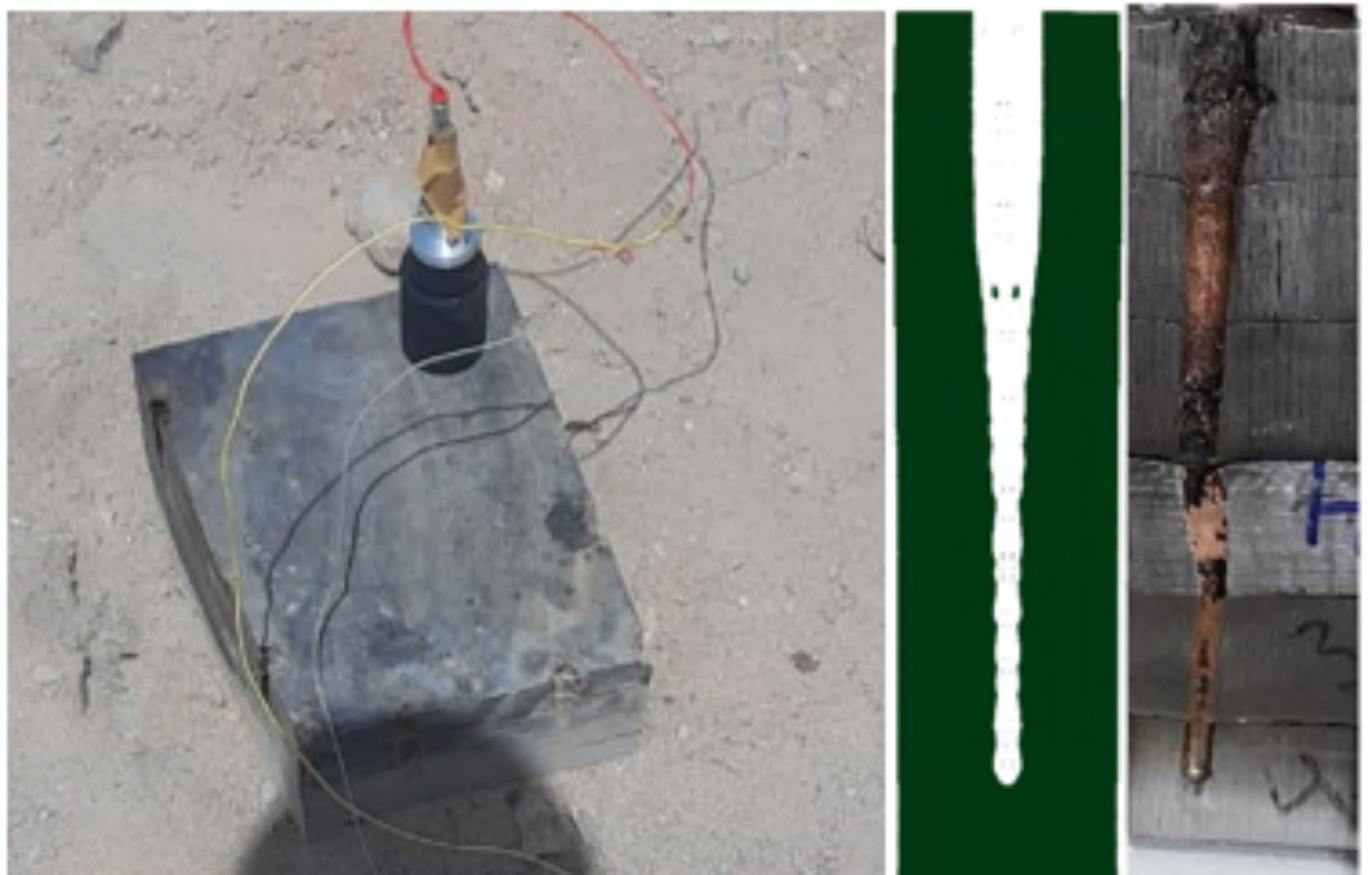
The penetration test setup (left), the real HMX-V5 shaped charge jet penetration crater profile (right) and numerical one (middle).

As an indicative measure to the shaped charge efficiency, the penetration capability into RHA targets is considered the factor of merit when some shaped charges are compared together from the penetration depth point of view. From the first impression, CL20-V5 explosive is expected to be the optimum one, however, other considerations should be considered such as cost and availability. These constrains makes the HMX-Viton is the best explosive when this research was conducted. Figure 18; right part shows the crater profile of the shaped charge loaded with HMX-V5 explosive, whereas the numerical penetration achieved with the same shaped charge is shown in the same Figure; middle. experimental penetration shows little variation w.r.t. the penetrated hole centreline, which may be attributed to the separation of the welded laminated RHA plates before doing the longitudinal section, which resulted in asymmetric hole profile. However, a general similarity between the numerical penetration modelling and the real experiment was demonstrated. The same scheme and the material model parameters were kept constant during the entire calculations of the jet penetrations into RHA targets using the different explosives.

**Fig. 19:**
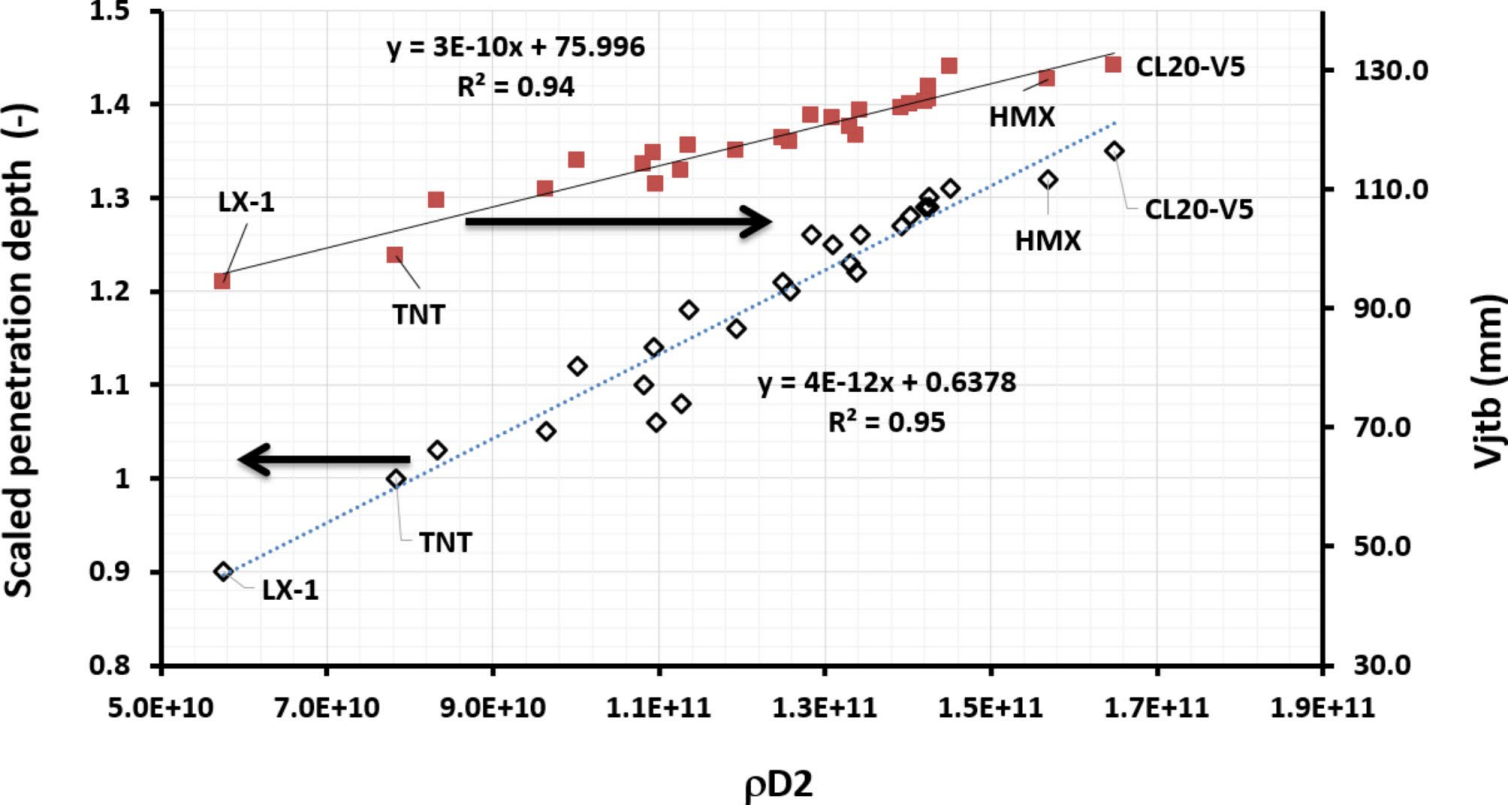
The dependence of the scaled penetration depth of shaped charges loaded with various explosive charges on the ρD^2^.

A linear relationship between the scaled depths of shaped charges penetration with the ρD^2^ value was observed in Fig. 19. This implies the importance of both the detonation velocity and the loading density together, which in turn directly has a direct impact on the detonation pressure of the used explosive and the accompanied energy imparted to the adjacent metallic liner. The effective jet length (the jet tip velocity multiplied by its breakup time) suggested by Held^47^ was found to form a direct criteria with both the ρD^2^ value and the relevant scaled penetration depth (considering the same constant densities (i.e.$\:\:\sqrt{({}_{j}⁄{}_{t})}$ and neglecting the little change in the cut-off velocity). This was confirmed with the SDM penetration model for the jet into RHA steel target materials including the three stages; the fully continuous, broken, fully broken-up jet fragments^48^.

Based on Fig. 17, the depth of penetration (DOP) can be approximated knowing the density and detonation velocity of the explosive charge as per formulae:

12 $$\:\frac{DOP,\:expl}{DOP,\:TNT}=\frac{4}{{10}^{12}}ρ{D}^{2}+0.6378\:.$$

VOD 8730 m/s for HMX-V5 at loading density of 1.84 g/cm^3^ .Therefore, the depth of penetration of the HMX-V5 equals about 1.1987 times that of the TNT explosive charge; i.e.

13 $${\text{DOP}}_{{({\text{EXP}}.)}} = {\text{1}}.{\text{1987 DOP}}_{{({\text{TNT)}}}},$$.

(13)

If the penetration depth of the TNT explosive charge is 12 cm (as an assumption), then the penetration depth of shaped charge loaded with HMX-V5 will be 14.38 cm. In our case, the experimental penetration is about 98% of this value, which suggests relatively satisfactory accuracy.

## Conclusions

The performance of 26 different explosive materials loaded to small calibre shaped charges have been assessed numerically using Autodyn where some of these tests have been verified experimentally. The jetting analysis showed large variation between the most powerful energetic CL-20 explosive and the least one; LX-1 in terms of the produced jet velocity (126% increase for CL-20 with respect to LX-1), jet mass percent (12.89% for LX-1 and 19.0% for CL-20), relevant kinetic energy (24 kJ and 51 kJ for LX-1 and CL-20; respectively) and the accompanied penetration depth into RHA steel targets (150% increase for CL-20 with respect to LX-1).

In this context, the jet tip velocity was validated using the VOD 812 apparatus by OZM research, with an error less than 4%. On the other hand, L3 flash x-ray radiograph with two head tubes of 300kv and 1Mv has been implemented to validate the jet symmetry, profile shape and its tip velocity, which showed only 2.1% different from that of numerical model and 1.47% different different from that using VOD 812 apparatus. Besides, the numerical SPH fragmentation algorithm showed remarkable increase in the fragment number and average fragment speed evolved from the shaped charge steel body when highly energetic explosive such as HMX, CL20 and BCHMX based explosives are used. Besides, the calculated average fragment speed has been validated when the high speed photography has revealed that the measured fragment speed has only 2.3% difference in comparison with the SPH calculations. In other words, the maximum average fragment speed of the most energetic explosive CL-20 has shown maximum value of 1555 m/s in comparison to 919 m/s for LX-1. The numerical penetration has been validated and verified using field testing against RHA steel target of an error less than 5%. Several empirical relationships have been proposed and proved the great influence of the explosives detonation velocities on the efficiency of shaped charges.

## Data Availability

The datasets used and/or analyzed during the current study available from the corresponding author on reasonable request.

## Abbreviations

PBX: Plastic bonded explosives
HMX: 1,3,5,7-tetranitro-1,3,5,7-tetrazocane
RDX: 1,3,5-trinitro-1,3,5-triazine
BCHMX: Cis-1,3,4,6-tetranitro-octahydro-imidazo-[4,5-d]imidazole
CL-20: 2,4,6,8,10,12-hexanitro-hexaazaisowurtzitane
TNAZ: 1,3,3-Trinitroazetidine
TNT: 2,4,6-trinitro-toluene
PETN: Pentaerythritol tetranitrate
Octol: Mixture of 70% HMX and 30% TNT
LX-14: 95.5% of HMX and 4.5% Estane R 5702-F1 binder
LX-19: 95.8% of CL-20 and 4.2% Estane 5703p binder
A-3: 91% RDX and 9% desensitizing wax
Viton A: Copolymer of hexafluoropropylene and vinylidene fluoride
RHA: Rolled homogeneous armor
EFP: Explosively formed penetrator
